# Genome-Wide Association Studies of Serum Magnesium, Potassium, and Sodium Concentrations Identify Six Loci Influencing Serum Magnesium Levels

**DOI:** 10.1371/journal.pgen.1001045

**Published:** 2010-08-05

**Authors:** Tamra E. Meyer, Germaine C. Verwoert, Shih-Jen Hwang, Nicole L. Glazer, Albert V. Smith, Frank J. A. van Rooij, Georg B. Ehret, Eric Boerwinkle, Janine F. Felix, Tennille S. Leak, Tamara B. Harris, Qiong Yang, Abbas Dehghan, Thor Aspelund, Ronit Katz, Georg Homuth, Thomas Kocher, Rainer Rettig, Janina S. Ried, Christian Gieger, Hanna Prucha, Arne Pfeufer, Thomas Meitinger, Josef Coresh, Albert Hofman, Mark J. Sarnak, Yii-Der Ida Chen, André G. Uitterlinden, Aravinda Chakravarti, Bruce M. Psaty, Cornelia M. van Duijn, W. H. Linda Kao, Jacqueline C. M. Witteman, Vilmundur Gudnason, David S. Siscovick, Caroline S. Fox, Anna Köttgen, The Genetic Factors for Osteoporosis (GEFOS) Consortium, Meta Analysis of Glucose and Insulin Related Traits Consortium (MAGIC)

**Affiliations:** 1 Human Genetics Center and Division of Epidemiology, The University of Texas Health Science Center at Houston, School of Public Health, Houston, Texas, United States of America; 2 National Cancer Institute, Division of Cancer Epidemiology and Genetics, Bethesda, Maryland, United States of America; 3 Department of Epidemiology, Erasmus MC, Rotterdam, The Netherlands; 4 Department of Internal Medicine, Erasmus MC, Rotterdam, The Netherlands; 5 The Netherlands Genomics Initiative–sponsored Netherlands Consortium for Healthy Aging (NGI-NCHA), Leiden, The Netherlands; 6 National Heart, Lung, and Blood Institute's Framingham Heart Study and the Center for Population Studies, Framingham, Massachusetts, United States of America; 7 Cardiovascular Health Research Unit and Department of Medicine, University of Washington, Seattle, Washington, United States of America; 8 Icelandic Heart Association, Kopavogur, Iceland; 9 University of Iceland, Reykjavik, Iceland; 10 McKusick-Nathans Institute of Genetic Medicine, Johns Hopkins University, Baltimore, Maryland, United States of America; 11 Division of Cardiology, Geneva University Hospital, Geneva, Switzerland; 12 Department of Epidemiology, Graduate School of Public Health, University of Pittsburgh, Pittsburgh, Pennsylvania, United States of America; 13 Laboratory of Epidemiology, Demography, and Biometry, National Institute on Aging, Baltimore, Maryland, United States of America; 14 Department of Biostatistics, Boston University School of Public Health, Boston, Massachusetts, United States of America; 15 Collaborative Health Studies Coordinating Center, University of Washington, Seattle, United States of America; 16 Interfaculty Institute for Genetics and Functional Genomics, University of Greifswald, Greifswald, Germany; 17 School of Dentistry, University of Greifswald, Greifswald, Germany; 18 Institute of Physiology, University of Greifswald, Greifswald, Germany; 19 Institute of Epidemiology, Helmholtz Zentrum München, Munich, Germany; 20 Institute of Human Genetics, Klinikum Rechts der Isar der TU München, Munich, Germany; 21 Clinic of Dermatology, Am Biederstein, Klinikum Rechts der Isar der TU München, Munich, Germany; 22 Institute of Human Genetics, Helmholtz Zentrum München, Munich, Germany; 23 Department of Epidemiology, Johns Hopkins University, Baltimore, Maryland, United States of America; 24 Division of Nephrology, Tufts Medical Center, Boston, Massachusetts, United States of America; 25 Medical Genetics Institute, Cedars-Sinai Medical Center, Los Angeles, California, United States of America; 26 Department of Clinical Chemistry, Erasmus MC, Rotterdam, The Netherlands; 27 Cardiovascular Health Research Unit, Departments of Medicine, Epidemiology, and Health Services, University of Washington, Seattle, Washington, United States of America; 28 Group Health Research Institute, Group Health Cooperative, Seattle, Washington, United States of America; 29 Division of Endocrinology, Brigham and Women's Hospital and Harvard Medical School, Boston, Massachusetts, United States of America; 30 Division of Nephrology, University Hospital Freiburg, Freiburg, Germany; Queensland Institute of Medical Research, Australia

## Abstract

Magnesium, potassium, and sodium, cations commonly measured in serum, are involved in many physiological processes including energy metabolism, nerve and muscle function, signal transduction, and fluid and blood pressure regulation. To evaluate the contribution of common genetic variation to normal physiologic variation in serum concentrations of these cations, we conducted genome-wide association studies of serum magnesium, potassium, and sodium concentrations using ∼2.5 million genotyped and imputed common single nucleotide polymorphisms (SNPs) in 15,366 participants of European descent from the international CHARGE Consortium. Study-specific results were combined using fixed-effects inverse-variance weighted meta-analysis. SNPs demonstrating genome-wide significant (p<5×10^−8^) or suggestive associations (p<4×10^−7^) were evaluated for replication in an additional 8,463 subjects of European descent. The association of common variants at six genomic regions (in or near *MUC1*, *ATP2B1*, *DCDC5*, *TRPM6*, *SHROOM3*, and *MDS1*) with serum magnesium levels was genome-wide significant when meta-analyzed with the replication dataset. All initially significant SNPs from the CHARGE Consortium showed nominal association with clinically defined hypomagnesemia, two showed association with kidney function, two with bone mineral density, and one of these also associated with fasting glucose levels. Common variants in *CNNM2*, a magnesium transporter studied only in model systems to date, as well as in *CNNM3* and *CNNM4*, were also associated with magnesium concentrations in this study. We observed no associations with serum sodium or potassium levels exceeding p<4×10^−7^. Follow-up studies of newly implicated genomic loci may provide additional insights into the regulation and homeostasis of human serum magnesium levels.

## Author Summary

Magnesium, potassium, and sodium are involved in important physiological processes. To better understand how common genetic variation may contribute to inter-individual differences in serum concentrations of these electrolytes, we evaluated single nucleotide polymorphisms (SNPs) across the genome in association with serum magnesium, potassium, and sodium levels in 15,366 participants of European descent from the CHARGE Consortium. We then verified the associations in an additional 8,463 study participants. Six different genomic regions contain variants that are reproducibly associated with serum magnesium levels, and only one of the regions had been previously known to influence serum magnesium concentrations in humans. The identified SNPs also show association with clinically defined hypomagnesemia, and some of them with traits that have been linked to serum magnesium levels, including kidney function, fasting glucose, and bone mineral density. We further provide evidence for a physiological role of magnesium transporters in humans which have previously only been studied in model systems. None of the SNPs evaluated in our study are significantly associated with serum levels of sodium or potassium. Additional studies are needed to investigate the underlying molecular mechanisms in order to help us understand the contribution of these newly identified regions to magnesium homeostasis.

## Introduction

Magnesium is the second most abundant intra-cellular cation and is a co-factor in several important reactions, including nucleic acid synthesis and many enzymatic reactions [1]. Nearly 60% of magnesium in the human body resides in bone, 20% in skeletal muscle, and 20% in soft tissue. Although only a fraction of total magnesium is present in blood, serum magnesium concentrations are reported to associate with several common and chronic diseases, including diabetes [2], hypertension [3], and osteoporosis [4]. Sodium and potassium are the most abundant cations in extra- and intracellular fluids, respectively [5], and are also commonly measured in serum. They have important roles in the maintenance of fluid and electrolyte balance as well as cell excitability.

Although most magnesium deficiencies are acquired [6], serum magnesium concentrations have been shown to have a heritable component with heritability estimates of ∼30% [7], [8]. In addition, several rare monogenic disorders have been identified that are characterized by abnormalities in magnesium homeostasis [1], [6], [9], including Gitelman syndrome (OMIM #263800), Bartter syndrome (OMIM #601678, #241200, #607364), and several hypomagnesemia syndromes (OMIM #602014, #154020, #248250, #611718, and #248190). Heritability estimates for serum sodium and potassium concentrations were comparable to the ones for magnesium in previous studies [10]–[13], and several monogenic diseases with disturbances in serum potassium or sodium concentrations exist [14].

Information on common genomic variants that are associated with serum cation concentrations in the general population may provide insights into physiologic regulators of electrolyte homeostasis. Thus, we undertook genome-wide association studies (GWAS) of serum magnesium, potassium and sodium concentrations in 15,366 subjects in the Cohorts for Heart and Aging Research in Genomic Epidemiology (CHARGE) Consortium. Since the kidney has an essential role in maintaining serum concentrations of these cations, and since magnesium, sodium, and potassium have been implicated in blood pressure regulation, we also assessed whether our newly identified variants were associated with glomerular filtration rate (eGFR) estimated from serum creatinine levels as a measure of kidney function as well as with systolic and diastolic blood pressure (SBP and DBP) in the CHARGE Consortium. We further evaluated the identified variants in association with fasting glucose in the Meta-Analyses of Glucose and Insulin Related Traits Consortium (MAGIC) [15] and bone mineral density (BMD) in the Genetic Factors for Osteoporosis (GEFOS) Consortium [16]; two continuous traits used to identify the presence of diabetes and osteoporosis.

## Results

Overall, 15,366 individuals of European descent from the Atherosclerosis Risk in Communities (ARIC) Study (N = 8,122), the Framingham Heart Study (FHS; N = 2,866), and the Rotterdam Study (RS; N = 4,378) contributed data to the discovery analyses of common variants associated with serum magnesium concentrations. Meta-analysis of serum sodium concentrations included information from 11,552 individuals from the three cohorts, and 13,683 individuals contributed information to the meta-analysis of serum potassium concentrations (including 3,370 participants from the Cardiovascular Health Study [CHS]). Selected characteristics for these four study samples as well as an additional CHARGE cohort that contributed information to secondary analyses of kidney function and blood pressure [The Age, Gene/Environment Susceptibility (AGES) —Reykjavik Study (N = 3,219)] are reported in Table 1.

**Table 1:** Baseline characteristics of the CHARGE Consortium discovery cohorts.*

	ARIC (N = 8,122)	FHS (N = 2,866)	RS (N = 4,378)	AGES (N = 3,219)	CHS (N = 3,370)
*N (%)*					
Men	3,831 (47)	1,372 (48)	1,690 (39)	1,352 (42)	1,339 (40)
Hypertension	2,201 (27)	575 (20)	2,478 (57)	1,127 (35)	1,533 (45)
Using hypertension medications	2,076 (26)	247 (9)	1,447 (26)	208 (6)	1,201 (36)
Hypomagnesemia (<0.7 mmol/L)	638 (8)	22 (1)	411 (9)	NA	NA
*Mean±SD*					
Age (y)	54±5.7	43±9.8	70±9.0	51±6.4†	72±5.4
BMI (kg/m^2^)	27±4.9	25±4.0	26±3.7	25±3.5	26±4.5
eGFR (ml/min/1.73 m^2^)	90±17.9	103±36.1	71±17.2	72±20.0†	80±22.6
SBP (mmHg)	119±17.0	121±16.0	139±22.1	132±16.9	135±21.0
DBP (mmHg)	72±10.0	78±9.6	74±11.7	83±9.6	70±11.4
Magnesium (mmol/L)	0.83±0.07	0.94±0.08	0.81±0.09	NA	NA

AGES, Age, Gene/Environment Susceptibility—Reykjavik Study; ARIC, The Atherosclerosis Risk in Communities Study; BMI, body mass index; CHARGE, Cohorts for Heart and Aging Research in Genomic Epidemiology; CHS, The Cardiovascular Health Study; DBP, diastolic blood pressure; eGFR, estimated glomerular filtration rate; FHS, The Framingham Heart Study; NA, not available; RS, The Rotterdam Study; SBP, systolic blood pressure; SD, standard deviation.

*Characteristics are reported for the population with magnesium concentrations for ARIC, FHS, and RS and for the population with information on blood pressure for CHS and AGES.

**†:** Age reported is at the time of blood pressure measurement. eGFR was measured at an average age of 75.

In total, 2,585,820 common single nucleotide polymorphisms (SNPs) were examined in association with serum magnesium, sodium, and potassium within each study, and the findings were meta-analyzed across studies using inverse-variance weighted fixed-effects models. SNPs were imputed in the individual studies as described in Table S1. No genome-wide significant (p<5×10^−8^) or suggestive (p<4×10^−7^) results were observed for serum sodium or potassium concentrations after adjustment for age, sex, and study center (where applicable). SNPs that showed evidence for association at p<1×10^−5^ after correction for genomic control are provided in Table S2 (sodium) and Table S3 (potassium). Q-Q plots of the observed versus expected p-value distributions for associations between the ∼2.5 million SNPs and magnesium, sodium and potassium levels are provided in Figure S1A, S1B, S1C. Heritability of serum magnesium, sodium, and potassium was estimated in the family-based FHS. Heritability was significant for serum magnesium (0.45; SE = 0.06; p = 1×10^−13^, N = 2,657) but not for serum sodium (0.04; SE = 0.06; p = 0.27, N = 2,416) or potassium (0.03; SE = 0.06; p = 0.29, N = 2,418) after excluding individuals on hypertension treatment. The traits were only weakly correlated in the ARIC study, the largest cohort in CHARGE (r^2^≤0.15).

**Figure 1:**
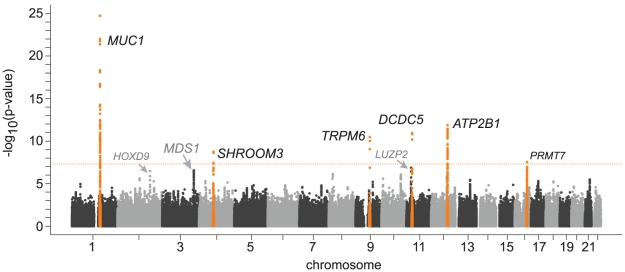
Genome-wide –log_10_(p-value) plot from association analyses with serum magnesium concentrations in 15,366 participants of European ancestry from the Cohorts for Heart and Aging Research in Genomic Epidemiology (CHARGE) Consortium. Adjusted for age, sex, and center.

Figure 1 shows the Manhattan plot for associations between SNPs and magnesium levels in the discovery cohorts after adjustment for age, sex, and center (where applicable). There were six regions with variants associated with serum magnesium concentrations at a genome-wide significance level of p<5×10^−8^. Information about the SNP with the lowest p-value within each region (lead SNP) is presented in Table 2; the lead SNPs were located in or near *MUC1* (91 kb region, chr 1), *SHROOM3 (*175 kb region, chr 4), *TRPM6* (77 kb region, chr 9), *DCDC5* (25 kb region, chr 11), *ATP2B1* (233 kb region, chr 12), and *PRMT7* (395 kb region, chr 16). Individually, the six genome-wide significant SNPs in the combined discovery and replication cohorts explained between 0.1 and 0.6% of the variance in serum magnesium concentrations; jointly, they explained about 1.6% of the variance (1.9% in the discovery cohorts and 1.2% in the replication cohorts). Three additional regions showed evidence of suggestive association (p<4×10^−7^) with serum magnesium concentrations (Table 2). Associations between the lead SNPs and serum magnesium within each of the discovery cohorts as well as their combined effect are presented in Table S4. Summary information for all SNPs associated with serum magnesium at p<10^−6^ is included in Table S5. Regional association plots for the six genomic regions with evidence for genome-wide association in the discovery cohorts are provided in Figure S2A, S2B, S2C, S2D, S2E, S2F. Results were similar when individuals on hypertension medications were excluded from the discovery analysis.

**Table 2:** Associations between serum magnesium levels and the lead regional genome-wide significant SNPs in the combined discovery (N = 15,366) and replication (N = 8,463) samples.

SNP	CHR	Location (base pairs)*	Function	CAF	Coded/Other Allele†	Closest Gene	% Variance Explained	Beta (mmol/L)‡	SE	P
*SNPs with genome-wide significant association after discovery (p<5×10^−8^)*										
**rs4072037**	**1**	**155162067**	**synonymous**	**0.46**	**C/T**	***MUC1***	**0.57**	**−0.010**	**0.001**	**2.01E**-**36**
**rs13146355**	**4**	**77412140**	**Intronic**	**0.56**	**G/A**	***SHROOM3***	**0.19**	**−0.005**	**0.001**	**6.27E**-**13**
**rs11144134**	**9**	**77499796**	**Intronic**	**0.92**	**T/C**	***TRPM6***	**0.23**	**−0.011**	**0.001**	**8.21E**-**15**
**rs3925584**	**11**	**30760335**	**Intergenic**	**0.45**	**C/T**	***DCDC5*** §	**0.25**	**−0.006**	**0.001**	**5.20E**-**16**
**rs7965584**	**12**	**90305779**	**Intergenic**	**0.29**	**G/A**	***ATP2B1*** §	**0.25**	**−0.007**	**0.001**	**1.05E**-**16**
rs7197653	16	68383047	Intronic	0.15	C/G	*PRMT7*	0.10	**−**0.005	0.001	2.02E-06
*SNPs with suggestive association after discovery (p<4×10^−7^)*										
rs2592394	2	176991779	Intergenic	0.30	A/G	*HOXD9* §	0.09	**−**0.004	0.001	4.61E-07
**rs448378**	**3**	**169100899**	**Intronic**	**0.47**	**G/A**	***MDS1***	**0.13**	**−0.004**	**0.001**	**1.25E-08**
rs4561213	11	24678819	Intronic	0.53	G/T	*LUZP2*	0.09	−0.004	**0.001**	2.60E-07

CHR, chromosome; CAF, coded allele frequency; SE, standard error; SNP, single nucleotide polymorphism.

*Location and distance to closest gene based on provisional NCBI Build 37.1.

**†:** Coded alleles are inversely associated with magnesium.

**‡:** Adjusted for age, sex, and study center (if applicable).

**§:** SNPs are located near the gene: rs3925584 is 126,151 bp from *DCDC5*, rs7965584 is 1,151,916 bp from *ATP2B1*and rs2592394 is 2,255 bp from *HOXD9*.

Replication of the lead SNPs with evidence of significant or suggestive association in the discovery cohorts was attempted in an additional 8,463 independent individuals of European descent (N = 1,641, KORA F3 Study; N = 1,809, KORA F4 Study; N = 4,065, SHIP Study; N = 948, ARIC Study). Mean serum magnesium levels in the replication cohorts were 0.83±0.07 (ARIC), 0.86±0.07 (KORA F3), 0.91±0.06 (KORA F4), and 0.78±0.09 (SHIP) mmol/L. At a Bonferroni-corrected significance level of 5.5×10^−3^ (0.05/9), five of the six SNPs with evidence of genome-wide significant association in the discovery samples showed evidence for replication in the independent replication cohorts (Table 2). Of the three SNPs with suggestive evidence for association in the discovery cohorts, the SNP at the *MDS1* locus showed evidence for independent replication, and combined with the discovery samples, reached a genome-wide level of significance (Table 2). Cohort-specific associations for the replication cohorts along with summary associations are presented in Table S6. Information about the quality of imputation for the lead SNPs within each cohort is reported in Table S7.

Replicated SNPs with evidence for genome-wide association in the discovery cohorts were related to clinically relevant hypomagnesemia, using a 0.7 mmol/L cutpoint [17]. All SNPs showed nominally significant p-values, and the odds ratios ranged from 1.11 (*SHROOM3*) to 1.27 (*MUC1*) per copy of the magnesium-lowering allele (Table 3).

**Table 3:** Association between hypomagnesemia, estimated glomerular filtration rate, fasting glucose, and bone mineral density with the lead replicated SNPs showing genome-wide significant associations with serum magnesium concentrations.

SNP	CHR	Closest Gene	N	Allele	OR*	95% CI	P
*Hypomagnesemia* †							
rs4072037	1	*MUC1*	12,500	C	1.27	1.16–1.39	3.92E-07
rs13146355	4	*SHROOM3*	12,500	G	1.11	1.01–1.21	3.30E-02
rs11144134	9	*TRPM6*	12,500	T	1.20	1.00–1.44	4.57E-02
rs3925584	11	*DCDC5*	12,500	C	1.18	1.08–1.29	3.22E-04
rs7965584	12	*ATP2B1*	12,500	G	1.24	1.13–1.37	6.38E-06

SNP	CHR	Closest Gene	N		Beta*	SE	P
*Estimated glomerular filtration rate (eGFR)* ‡							
rs4072037	1	*MUC1*	21,805	C	1.31E-04	0.002	9.54E-01
rs13146355	4	*SHROOM3*	21,805	G	0.015	0.002	4.28E-11
rs11144134	9	*TRPM6*	21,805	T	−1.79E-04	0.004	9.68E-01
rs3925584	11	*DCDC5*	21,805	C	0.009	0.002	4.11E-05
rs7965584	12	*ATP2B1*	21,805	G	0.004	0.002	1.32E-01
*Fasting glucose* §							
rs4072037	1	*MUC1*	40,496	C	−0.012	0.004	3.58E-03
rs13146355	4	*SHROOM3*	45,727	G	3.00E-04	0.004	9.33E-01
rs11144134	9	*TRPM6*	28,137	T	0.006	0.010	5.30E-01
rs3925584	11	*DCDC5*	46,180	C	−0.001	0.004	8.89E-01
rs7965584	12	*ATP2B1*	46,171	G	0.002	0.004	6.69E-01
*Femoral Neck bone mineral density (BMD)* ∥							
rs4072037	1	*MUC1*	19,195	C	0.039	0.012	1.17E-03
rs13146355	4	*SHROOM3*	19,195	G	0.008	0.012	4.92E-01
rs11144134	9	*TRPM6*	19,195	T	0.076	0.025	2.00E-03
rs3925584	11	*DCDC5*	19,195	C	−0.006	0.012	5.84E-01
rs7965584	12	*ATP2B1*	19,195	G	−0.007	0.013	5.80E-01

CHR, chromosome; CI, confidence interval; OR, odds ratio; SE, standard error; SNP, single nucleotide polymorphism.

*Adjusted for age, sex, and study center (if applicable); the association reported is for the magnesium-lowering allele.

**†:** Includes the following CHARGE cohorts: ARIC (N = 8,122), RS (N = 4,378); OR is from logistic regression of hypomagnesemia (<0.7 mmol/L).

**‡:** Includes the following CHARGE cohorts: AGES (N = 3,219), ARIC (N = 8,069), CHS (N = 3,282), FHS (N = 2,861), RS (N = 4,374); beta is for natural log transformed eGFR (ml/min/1.73 m^2^).

**§:** Results are from meta-analyses of up to 46,180 individuals from 21 MAGIC cohorts in non-diabetics of European ancestry; beta is for each unit increase in fasting glucose (mmol/L) and is additionally adjusted for geographic covariates (where applicable) and age squared (FHS only).

**∥:** Results are from meta-analyses of 19,195 individuals from five cohorts in the GEFOS consortium; beta is for per allele-copy change of BMD as measured in standard deviations from the population mean and is adjusted for age, weight, sex, and study. Associations with lumbar spine BMD were similar to femoral neck BMD.

As the kidney is one of the primary regulators of serum magnesium concentrations, we also examined these SNPs in association with the kidney function measure, eGFR. The allele associated with lower magnesium levels at two of the SNPs showed evidence for association with higher eGFR: rs3925584 near *DCDC5* (p = 4.1×10^−5^) and rs77412140 in *SHROOM3* (p = 4.3×10^−11^; Table 3). Adjusting the magnesium-SNP associations for eGFR did not materially change the association with serum magnesium levels (eGFR-adjusted beta = −0.006, p = 6.3×10^−12^ for the *DCDC5* and beta = −0.005, p = 9.5×10^−9^ for the *SHROOM3* SNP).

Since magnesium levels associate with hypertension [3], diabetes [2], and osteoporosis [4] in observational studies, we further evaluated the lead replicated SNPs in association with the continuous traits used to define these chronic conditions: SBP and DBP (CHARGE cohorts), fasting glucose (MAGIC Consortium [15]) and BMD (GEFOS Consortium [16]). None of the SNPs was associated with SBP or DBP in our study (Table S8), but the allele associated with lower magnesium levels at the *MUC1* SNP showed nominally-significant evidence of association with lower fasting glucose after correcting for the number of SNPs investigated (Bonferroni-corrected significance level = 0.05/5 = 0.01; Table 3). The same allele showed association with higher BMD, as did the magnesium-lowering allele of the *TRPM6* SNP (Table 3).

Finally, in the CHARGE discovery cohorts, we evaluated genes that contain rare variants known to cause monogenic syndromes of abnormal magnesium metabolism [6] for common susceptibility variants that associate with normal magnesium levels. We also evaluated common SNPs in genes that have been implicated as magnesium transporters in model systems [18] but, to date, have an unknown functional role in humans from the general population. The number of SNPs per gene examined as well as summary information for the SNP with the lowest p-value from each gene are provided in Table 4. Common variants in *CNNM2* (rs3740393, p = 8.6×10^−7^), *CNNM3* (rs994430, p = 1.5×10^−4^) and *CNNM4* (rs6746896, p = 7.0×10^−5^) were associated with magnesium concentrations after applying a Bonferroni-correction for the number of SNPs examined in each region.

**Table 4:** SNPs in or near genes known to cause monogenic syndromes with abnormal magnesium metabolism or near known magnesium transport genes in association with serum magnesium in 15,366 participants from the CHARGE Consortium.

Gene	# of SNPs†	Lead SNP	CAF	Coded/Other Allele	CHR	Location (base pairs)	Beta (mmol/L)‡	SE	P	
*Mendelian Genes*										
*CLCNKB*	108	rs848305	0.89	T/A	1	16316825	−0.007	0.002	2.74E-03	
*CLDN19*	62	rs719676	0.76	A/G	1	43254108	−0.002	0.001	5.51E-02	
*BSND*	174	rs9326034	0.01	A/G	1	55530664	−0.018	0.006	5.18E-03	
*CASR*	261	rs17251221	0.86	A/G	3	121993247	−0.004	0.001	1.31E-03	
*CLDN16*	213	rs9990270	0.42	C/G	3	190176606	−0.002	9.00E-04	2.58E-02	
*EGF*	255	rs11569033	0.96	A/G	4	110905803	−0.013	0.004	1.39E-03	
*FXYD2*	165	rs948100	0.09	G/A	11	117660275	−0.003	0.002	3.65E-02	
*KCNJ1*	190	rs496844	0.49	G/C	11	128650029	−0.002	9.00E-04	2.49E-03	
*SLC12A1*	100	rs17428448	0.01	G/T	15	48551594	−0.049	0.022	2.62E-02	
*SLC12A3*	195	rs8048695	0.02	A/G	16	56910986	−0.023	0.007	7.64E-04	
*Magnesium Transport Genes* §										
*SLC41A1*	117	rs17348507	0.05	G/A	1	205814735	−0.004	0.002	5.39E-02	
*CNNM4*	42	rs6746896	0.66	A/G	2	97410949	−0.004	0.001	7.01E-05	
*CNNM3*	46	rs994430	0.59	A/T	2	97439001	−0.004	9.00E-04	1.53E-04	
*SLC41A3*	277	rs7617162	0.35	T/C	3	125755450	−0.002	9.00E-04	2.03E-02	
*NIPAL1 (NPAL1)*	100	rs17470528	0.83	T/C	4	48052003	−0.003	0.001	1.73E-02	
*NIPAL4 (ICHTHYIN)*	124	rs11134799	0.11	C/T	5	156958290	−0.003	0.001	5.68E-02	
*BTBD9*	536	rs4714146	0.91	G/A	6	38287910	−0.006	0.002	5.00E-04	
*ZDHHC13*	228	rs7116312	0.27	G/A	7	19079292	−0.004	0.001	5.31E-04	
*TUSC3*	557	rs12681567	0.10	A/C	8	15657603	−0.003	0.002	2.35E-02	
*CNNM1*	211	rs2490281	0.97	G/C	10	101192482	−0.006	0.003	3.92E-02	
*CNNM2*	222	rs3740393	0.86	G/C	10	104636655	−0.006	0.001	8.58E-07	
*ZDHHC17*	170	rs11115332	0.96	C/G	12	77171192	−0.004	0.002	8.65E-02	
*SLC41A2*	197	rs2463021	0.88	A/T	12	105168431	−0.003	0.002	3.99E-02	
*NIPA1*	123	rs4778439	0.95	G/A	15	23002053	−0.005	0.002	3.23E-02	
*NIPA2*	147	rs4778439	0.95	G/A	15	23002053	−0.005	0.002	3.23E-02	
*TRPM7*	177	rs2163098	0.03	G/C	15	50906498	−0.005	0.003	6.01E-02	

CAF, coded allele frequency; SE, standard error; SNPs, single nucleotide polymorphisms.

SNP locations are based on the provisional NCBI Build 37.1.

**†:** Within 60 kb of the gene.

**‡:** Adjusted for age, sex, and study center (if applicable); the association reported is for the magnesium-lowering allele and p-values are adjusted for genomic control. Proportion of variance in serum magnesium levels explained by the listed SNPs ranged from 0.11% by rs17251221 to <0.001.

**§:** *MAGT1* and *MMGT1*were not investigated because of their localization on the X chromosome.

## Discussion

We report a large genome-wide association study of serum magnesium, potassium and sodium levels in 15,366 community-dwelling subjects of European ancestry from the CHARGE Consortium. Associations with serum potassium and sodium did not reach the level of genome-wide significance in our study, but common genetic variants in six genomic regions in or near the *MUC1*, *MDS1, SHROOM3, TRPM6*, *DCDC5*, and *ATP2B1* genes were significantly and reproducibly associated with serum magnesium levels and clinically defined hypomagnesemia. Together, these SNPs explained about 1.6% of variation in serum magnesium levels. Variation at the *DCDC5* SNP (rs3925584) on chromosome 11 and the *SHROOM3* SNP (rs13146355) on chromosome 4 was also independently associated with eGFR, a measure of kidney function, while the *MUC1* SNP (rs4072037) was associated with fasting glucose as well as BMD and the *TRPM6* (rs11144134**)** SNP was associated with BMD. Finally, we provide evidence for a role of the magnesium transporters encoded by *CNNM2* as well as *CNNM3* and *CNNM4* in the regulation of physiological magnesium homeostasis in humans.

Magnesium homeostasis is maintained as a balance between intestinal magnesium absorption and renal magnesium excretion [7]. Magnesium transport in the kidney occurs both by passive paracellular reabsorption in the loop of Henle and by active transcellular reabsorption in the distal convoluted tubule [7].

Of the loci discovered here, only *TRPM6* on chromosome 9 had a previously known role in magnesium homeostasis. *TRPM6* encodes a TRP ion channel subunit, which is abundantly expressed in the gut and the kidney [19], [20], where it is responsible for transcellular magnesium transport by mediating magnesium reuptake at the apical membrane of renal epithelial cells in the distal tubule [9]. Rare mutations in *TRPM6* are a cause of autosomal recessive hypomagnesemia with secondary hypocalcemia (OMIM #602014) [19], [20]. The common T allele of rs11144134 in *TRPM6* that associated with lower serum magnesium levels in our study was also associated with higher femoral neck and lumbar spine BMD. Although magnesium-deficiency has been linked to osteoporosis and low BMD in observational and animal studies [9], our observations are in line with the higher BMD observed in patients with low plasma magnesium levels as a result of Gitelman's syndrome [21].

On chromosome 12, we identified variants in the *ATP2B1* gene region as associated with serum magnesium concentrations. This gene encodes plasma-membrane calcium ATPase 1 (PMCA1) [22], responsible for the removal of calcium ions from cells. One previous study reported that the phosphatase activity of PMCA1 is dependent on magnesium ions [23]. While magnesium uptake via TRPM6 at the apical membrane of epithelial cells has been demonstrated as the mechanism for magnesium entry, the mechanism by which magnesium ions exit the cells at the basolateral membrane is hitherto unknown [9]. Our epidemiologic findings relating variation in *ATP2B1* to serum magnesium concentrations, combined with the localization of PMCA1 in the basolateral membrane of epithelial cells in the distal renal tubule, makes PMCA1 an interesting candidate for further functional studies of renal magnesium transport. The genomic region containing the *ATP2B1* gene was previously identified in a genome-wide association study of blood pressure and hypertension [24]. Linkage disequilibrium (LD) between the blood pressure associated variant and the one reported in our study is low (r^2^ = 0.013 in HapMap CEU), supporting the independent effects of the two variants on blood pressure and magnesium homeostasis.

The genomic region containing the *SHROOM3* gene on chromosome 4 has been associated with eGFR in a previous GWAS [25] and with serum creatinine in another large consortium study [26]. Previously described eGFR/creatinine-associated variants are in strong LD with the magnesium-associated one reported in our study (r^2^>0.8 in HapMap CEU), in agreement with the significant association with eGFR detected in our study (p = 4.3×10^−11^). The magnitude of the association between the SNP and magnesium levels remained unchanged after adjustment for eGFR, which may suggest a pleiotropic effect of the same underlying causal variant.

The region on chromosome 1 spans about 100 kb and contains many genes. The SNP with the strongest association within this region was located in the gene *MUC1*. *MUC1* encodes mucin 1, a membrane bound, glycosylated phosphoprotein. It is attached to the apical surface of many epithelia, where it binds pathogens and functions in a cell signaling capacity. Aberrant forms of the protein have been associated with carcinomas. In addition to the observed association with lower serum magnesium levels, the C allele at rs4072037 in *MUC1* was also associated with higher femoral neck and lumbar spine BMD as well as with lower fasting glucose levels in two large consortia. The direction of association with BMD is consistent with the one we observed for the magnesium-lowering allele of rs11144134 in *TRPM6*.

The closest gene to the associated SNP on chromosome 11, rs3925584, is doublecortin domain containing 5 (*DCDC5*) of currently unknown physiological function. LD in the region also extends to the neighboring *MPPED2* gene, which encodes for a metallophosphoesterase that needs divalent metal ions for its catalytic activity [27]. Variants in the *DCDC5* genetic region were identified in a large GWAS as associated with lumbar spine BMD [16]. Although the reported BMD-associated variant and the variant associated with serum magnesium in our study are only in low LD (r^2^ = 0.04 in HapMap CEU), it is of interest that variation in three regions identified in our study (*MUC1*, *TRPM6*, *DCDC5*) can be linked to measures of BMD. In addition, the SNP we identified near *DCDC5* also showed some association with eGFR, and the association with magnesium levels remained unchanged upon adjustment for eGFR.

Finally, the rs448378 SNP on chromosome 3 is located in the myelodysplasia syndrome 1 (*MDS1*) gene. Like *MUC1* and *DCDC5*, *MDS1* is not an obvious candidate for magnesium homeostasis based on prior biological knowledge.

Previous studies in model systems have identified several genes coding for magnesium transport proteins [7], [28]–[34], but the contribution of common genetic variation in these genes to magnesium homeostasis in humans is unclear. Common variants in *CNNM2*, *CNNM3*, and *CNNM4* showed significant association with serum magnesium concentrations in our study after applying a conservative Bonferroni correction for the number of regional SNPs investigated, supporting the role of these proteins in human magnesium homeostasis under physiological conditions. The *CNNM2*-encoded magnesium transporter, ACDP2, belongs to the ancient conserved domain proteins (ACDP) family [35]. It is widely expressed in human tissues with strongest levels in brain, kidney and placenta [35], and experimental studies provide evidence for its involvement in magnesium transport [29], [36]. Rare mutations in *CNNM4* have recently been reported as a cause of autosomal-recessive cone-rod dystrophy with amelogenesis imperfecta [37], [38]. A magnesium transport function of the encoded ACDP4 has not yet been shown. Little is known about ACDP3 encoded by *CNNM3*; due to the close physical proximity of *CNNM3* and *CNNM4*, common variants associated with magnesium concentrations in our study may not represent independent signals. We further identify several common variants in genes responsible for monogenic disorders of magnesium metabolism that show some degree of association with serum magnesium concentrations in our study. As true associations may be missed at the stringent significance levels applied in genome-wide association studies, we noted the SNP with the lowest p-value in each of the genetic regions although they did not show evidence of genome-wide significant association. These results should therefore be interpreted with caution; on the other hand, applying a region-wide Bonferroni-correction as we did for these candidate regions may be overly conservative due to the presence of linkage disequilibrium.

There are several potential explanations for the observed lack of genome-wide evidence of associations with serum potassium or sodium levels in our study. While we had based our decision to conduct GWAS of serum sodium and potassium levels on earlier point estimates for heritability on the order of 25–30%, we only observed significant heritability for serum magnesium levels but not for serum levels of sodium or potassium. These findings are not necessarily inconsistent with previous estimates since earlier studies were mostly small and the 95% confidence intervals of heritability estimates for either serum sodium or potassium concentrations or both included 0. Other potential explanations for the difference in heritability estimates include our exclusion of individuals on hypertension medication, differences in the statistical model, and differences in study sample characteristics. Another reason for the lack of findings could be that genetic variants other than common SNPs could be of importance, which could not be detected in our study. Finally, fewer individuals were available for the analyses of serum sodium and potassium concentrations compared to magnesium concentrations thus impacting statistical power. The weak correlation of serum magnesium with serum sodium and potassium levels we observed (r^2^≤0.15) is consistent with the identification of genomic regions specific to serum magnesium.

Several limitations of this study should be considered when interpreting the results. Serum magnesium concentrations represent only a small portion of the total magnesium stores in the body, and markers associated with serum magnesium concentrations are therefore not necessarily markers of total magnesium stores. Second, results from our study are based on individuals of European descent only and should be replicated in other ethnicities. Third, our study likely did not have sufficient power to detect common variants in association with serum sodium or potassium concentrations. Finally, the functional significance of the lead SNPs identified in this study is unknown, and true causal variants likely remain to be determined. The proportion of serum magnesium variance explained by the SNPs identified here is modest, as has been observed from GWAS of other traits [39]. However, the genes discovered in our study provide a basis for future studies of magnesium homeostasis and for the targeted investigation of the presence of rare genetic variants of larger effect.

In conclusion, we identified six genomic regions that contained common variants reproducibly associated with serum magnesium levels in a genome-wide meta-analysis of CHARGE cohorts and four independent replication cohorts. All of the variants were nominally associated with clinically defined hypomagnesemia, and lead SNPs in four of the regions were also associated with measures of kidney function, fasting glucose, and BMD. As only the *TRPM6* gene was previously known to be involved in magnesium homeostasis, follow-up of the other associated regions may provide additional clues to the regulation of magnesium homeostasis in humans.

## Materials and Methods

### Ethics statement

Each of the cohorts collected written informed consent from study participants and received approval from their respective Institutional Review Boards.

### Discovery study samples

The CHARGE Consortium was established to facilitate meta-analysis of GWAS for traits related to cardiovascular disease (CVD) and aging [40]. Briefly, five large, population-based cohort studies from the United States and Europe with genome-wide genotyping information available in 2007 to 2008 were included: AGES—Reykjavik, ARIC, CHS, FHS, and RS. Detailed information about each cohort is provided in other references (AGES—Reykjavik [41]; ARIC [42]; CHS [43]; FHS [44]–[47]; RS [48]) and is summarized below.

The AGES—Reykjavik Study includes a sample of 5,764 survivors from the Reykjavik Study of 30,795 men and women born between 1907 and 1935. The ARIC study includes 15,792 men and women aged 45 to 64 who were enrolled in a prospective follow-up study from four US communities from 1987 to 1989. The CHS includes 5,201 mostly Caucasian participants aged 65 years or older that were randomly sampled from Medicare lists in four US communities from 1989 to 1990. The FHS recruited 5,209 participants aged 28 to 62 from Framingham, Massachusetts beginning in 1948. Beginning in 1971, 5,124 offspring of the original cohort members and the offspring's spouses were also recruited as part of the Offspring Cohort. FHS subjects in this study are from the Offspring Cohort who attended the second examination in 1971–1973. Finally, the RS recruited 7,983 subjects aged 55 years or older from Ommoord, a suburb of Rotterdam, between 1990 and 1993. Only subjects self-reporting European ancestry from each cohort are included as a part of this study.

### Replication study samples

#### ARIC Study

After conducting the meta-analysis within the CHARGE Study, genotype data on an additional 948 study participants of European ancestry became available within ARIC. These individuals were independent from the ones included in the discovery sample: they were not part of the discovery, had no first-degree relationship with any individual in the discovery sample, and would not have been classified as an outlier based on allele sharing measures generated during quality control procedures of the discovery sample.

#### KORA F3 and F4

The KORA Study is a series of independent population-based epidemiological surveys of participants living in the region of Augsburg, Southern Germany [49]. All survey participants were residents of German nationality identified through the registration office and were examined in 1994/95 (KORA S3) and 1999/2001 (KORA S4). In 2004/05, 3,006 subjects participated in a 10-year follow-up examination of S3 (KORA F3) and in 2006/08, 3,080 subjects participated in a 7-year follow-up examination of S4 (KORA F4). Individuals for genotyping in KORA F3 and KORA F4 were randomly selected. The age range of the participants was 25 to 74 years at recruitment.

#### SHIP

The Study of Health in Pomerania (SHIP) is a cross-sectional survey in West Pomerania, the north-east area of Germany [50]. A sample from the population aged 20 to 79 years was drawn from population registries. Only individuals with German citizenship and main residency in the study area were included. Of 7,008 subjects sampled, 4,310 participants comprised the final SHIP population.

### Genotyping and imputation

Details of genotyping methods, exclusion criteria, and imputation methods for the discovery and replication samples can be found in Table S1. Briefly, SNPs were genotyped within each cohort from 2006–2008 using commercially available whole-genome platforms, and each cohort imputed genotypes to a common set of about 2.5 million autosomal SNPs. Imputation was carried out using MACH version 1.09/.15/.16 (AGES—Reykjavik, ARIC, FHS, KORA and RS) (accessed from http://www.sph.umich.edu/csg/abecasis/MACH/), BimBam version 0.99 [51] software (CHS), or IMPUTEv0.5.0 [52] (SHIP). For the imputation, genotype data from the individual studies was combined with genotype data from HapMap CEU samples to probabilistically infer the allelic dosage for each SNP (a fractional value from 0.0 to 2.0) based on the HapMap CEU haplotype structure. Imputation quality scores were calculated for each SNP as the ratio of observed dosage-variance to the expected binomial variance.

### Study variables

The primary outcomes for this study were serum concentrations of magnesium, potassium and sodium. We additionally evaluated the lead SNPs identified in association with other clinically-relevant phenotypes, including hypomagnesemia (defined as serum magnesium <0.7 mmol/L; CHARGE), blood pressure (CHARGE), eGFR (CHARGE) fasting glucose (MAGIC), and BMD (GEFOS). Serum magnesium (discovery: ARIC, FHS, RS; replication: ARIC, KORA F3, KORA F4, SHIP), sodium (ARIC, FHS, RS), and potassium (ARIC, CHS, FHS, RS) concentrations were measured using standard protocols from fasting blood, where possible. Serum magnesium levels were determined using the method described by Gindler and Heth with metallochromic dye, Calmigate [1,-[1-hydroxy-4-methyl-2-phenylazo)-2-napthol-4-sulfonic acid] in the ARIC Study, by METPATH in FHS, with a Merck Diagnostica kit (method Xylidyl blue) on an Elan Autoanalyzer (Merk) in RS, with a Xylidylblue kit on a Modular analyzer (Roche) in the KORA Study, or using a commercial colorimetric test (Roche Diagnostics, Mannheim, Germany) with a Hitachi 717 autoanalyzer in the SHIP Study. Sodium and potassium levels were measured using standard ion electrode devices in all cohorts.

Detailed descriptions of blood pressure and eGFR traits are given in other references [24], [25] and are described in brief here. Serum creatinine, used to calculate eGFR, was measured using a modified kinetic Jaffe method (ARIC, CHS, FHS, RS) or an enzymatic method (AGES—Reykjavik). Creatinine values were calibrated to age- and sex-adjusted mean values from a nationally representative study as described previously [53], and eGFR (ml/min/1.73 m^2^) was calculated using the 4-variable MDRD Study formula [54]. Due to the skewed distribution, a natural log transformation was applied before the association analyses. Repeated resting SBP and DBP measures were recorded by trained staff in all studies, and the average of multiple readings was used. Height and weight were measured by trained study personnel in all studies and were used to calculate BMI (kg/m^2^). Use of blood pressure medications was defined differently in the different cohorts, but for all cohorts, hypertension medication use was determined at the time of serum electrolyte determination and included all classes of anti-hypertension medications commonly prescribed at the time, including beta-blockers, diuretics, ACE-inhibitors, angiotensin type-2 antagonists, calcium-channel blockers, as well as combination therapies.

### Statistical analysis

SNPs were modeled as allelic dosages in all analyses. Genome-wide analyses of electrolyte concentrations (magnesium, potassium, and sodium) were conducted within the R package ProbABEL (http://mga.bionet.nsc.ru/~yurii/ABEL/) [55] for ARIC, CHS and RS, or using linear mixed effects regression models in the R kinship package to account for pedigree structure in FHS. SNP-electrolyte associations were adjusted for age, sex, and study center, where applicable. For analyses of sodium and potassium concentrations subjects using any hypertension medications at the time of electrolyte assessment were excluded to avoid a possible influence of the medications on serum concentrations of sodium and potassium. Genomic control correction based on median chi-square was used within each study to adjust for inflation of the test statistics prior to meta-analysis, as well as applied to the combined results after the meta-analysis. Inverse-variance weighted fixed-effects meta-analyses were carried out by two independent analysts using the software METAL (www.sph.umich.edu/csg/abecasis/metal/) for the ∼2.5 million SNPs across ARIC, FHS, RS, and CHS (potassium only). After meta-analysis, results were filtered to remove SNPs with low minor allele frequency (<0.01). Statistical heterogeneity was evaluated using Cochrane's *χ^2^* test (Q-test). P-values <5×10^−8^ were used to indicate genome-wide significant results. The size of the associated regions was determined using the positions of the most upstream and downstream regional SNPs with p-values<5×10^−5^. Manhattan and Q-Q plots were generated for the meta-analyzed data using the R statistical software package (http://www.R-project.org). Plots of the –log_10_(p-values) by genomic position for associations within regions of statistical significance were generated using the SNAP program (http://www.broad.mit.edu/mpg/snap/ldsearch.php). In SNAP, the HapMap CEU population was used as the reference group to map LD patterns. In the family-based FHS, heritability of serum magnesium, sodium, and potassium was estimated using age and sex-adjusted residuals in a variance components model that estimated additive genetic heritability and a random environmental component using SOLAR v.1.4 [56].

The six lead SNPs with evidence of genome-wide significant association in discovery plus an additional three SNPs with suggestive evidence of association were evaluated for independent replication. In the replication studies, SNP-magnesium associations were determined in linear regression models as described for the discovery cohorts. Inverse-variance weighted fixed effects meta-analysis was used to determine associations across the replication samples and to calculate the overall combined associations for the discovery and replication cohorts.

For the lead SNPs, we calculated the percent of magnesium variance attributable to the SNP as the difference in the adjusted r^2^ value for a model containing the SNP, age, sex, and study center, where applicable, to a model containing only age, sex and study center, expressed as a percent. Assuming independent effects of the SNPs, we added the individual variance across the SNPs to calculate the total variance explained by the set of SNPs. The independence assumption was verified by simultaneous inclusion of all SNPs into a regression model. We also evaluated the five lead SNPs from CHARGE with evidence for replication in logistic models of hypomagnesemia (in ARIC and RS only because of small numbers of subjects with hypomagnesemia in FHS) or linear models of eGFR (ml/min/1.73 m^2^) adjusted for age, sex, and study center. Results for blood pressure (mm Hg) traits in association with the SNPs were adjusted for age, age squared, sex, and BMI to be consistent with the published data from a GWAS of blood pressure in the CHARGE Consortium, and blood pressure among treated and untreated individuals was modeled as described in this publication [24], [57]. Inverse-variance weighted fixed effects meta-analysis was used to determine summary effect estimates for these additional traits.

We further evaluated these SNPs in association with fasting glucose and BMD as an *in silico* lookup in large available datasets from two consortia. Fasting glucose associations were available from up to 46,180 subjects of European descent from the MAGIC Consortium [15], and BMD associations (femoral neck and lumbar spine) in 19,195 subjects of Northern European descent from the GEFOS Consortium [16].

To examine the association between serum magnesium levels and common variation in previously identified magnesium transporter proteins from model systems [7], [28]–[34] or in genes with rare variants responsible for monogenic disorders of magnesium metabolism, we examined associations with SNPs within 60 kb of the genes [58] and reported the association and annotation for the lead SNP within each gene region.

## Supporting Information

Figure S1 Q-Q plots showing the distribution of observed versus expected −log_10_(p-values) for the meta-analyses of magnesium (A), sodium (B), and potassium (C) in the CHARGE Consortium.
(0.09 MB TIF)

Figure S2 Regional association plots for SNPs and serum magnesium concentrations in 15,366 white participants from the CHARGE Consortium. Figures show −log_10_(p-values) by chromosomal position around the magnesium-associated regions along with any recombination hotspots in HapMap CEU. Genes that map within the regions are also noted on the plots. (A) SNPs in *MUC1* region; (B) SNPs in *ATP2B1* region; (C) SNPs in *DCDC5* region; (D) SNPs in *TRPM6* region; (E) SNPs in *SHROOM3* region; (F) SNPs in *PRMT7* region.
(0.17 MB TIF)

Table S1 Study-specific genotyping and imputation information for discovery and replication studies.
(0.04 MB DOC)

Table S2 SNP associations with serum sodium concentrations at p<10^-5^ in the CHARGE cohorts.
(0.06 MB DOC)

Table S3 SNP associations with serum potassium concentrations at p<10^-5^ in the CHARGE cohorts.
(0.03 MB DOC)

Table S4 Study-specific associations for magnesium levels and the lead regional magnesium genome-wide association study hits in the discovery cohorts.
(0.05 MB DOC)

Table S5 SNP association with serum magnesium concentrations at p<10^-6^ in the CHARGE cohorts.
(0.46 MB DOC)

Table S6 Study-specific associations for magnesium levels and the lead regional magnesium genome-wide association study hits in the replication cohorts.
(0.05 MB DOC)

Table S7 Imputation quality for SNPs with significant and suggestive association with serum magnesium concentrations.
(0.04 MB DOC)

Table S8 Association between systolic and diastolic blood pressure with the lead replicated SNPs showing genome-wide significant associations with serum magnesium concentrations in the CHARGE Consortium.
(0.04 MB DOC)

Text S1 Supporting information.
(0.10 MB DOC)
