# Supplementary figures and images for: Genome-Wide Association Studies of Serum Magnesium, Potassium, and Sodium Concentrations Identify Six Loci Influencing Serum Magnesium Levels
Source: PLoS Genet. 2010 Aug 5;6(8):e1001045. doi: 10.1371/journal.pgen.1001045 (PMC2916845; doi:10.1371/journal.pgen.1001045)

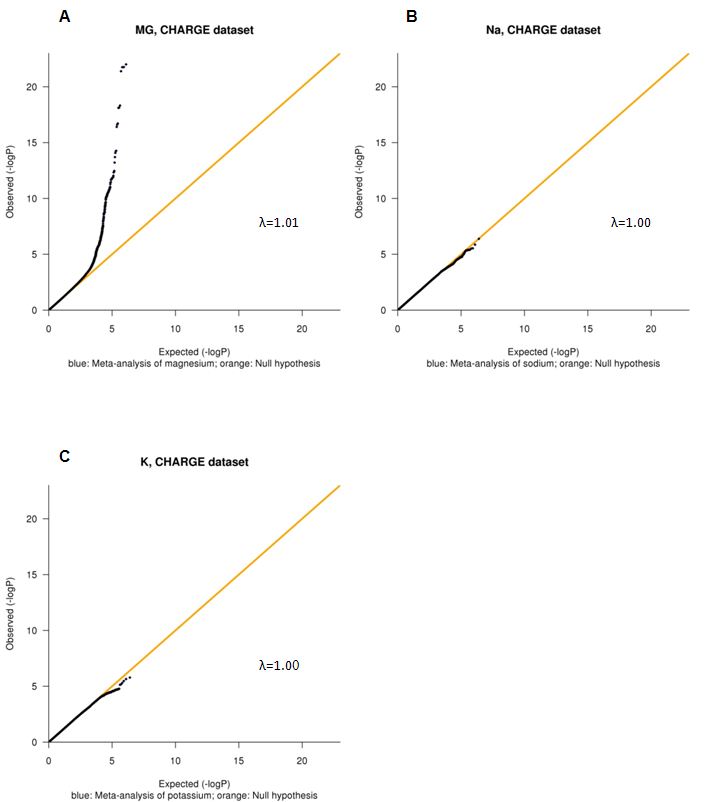

Supplement: Figure S1 — Q-Q plots showing the distribution of observed versus expected −log10(p-values) for the meta-analyses of magnesium (A), sodium (B), and potassium (C) in the CHARGE Consortium. (0.09 MB TIF) [file pgen.1001045.s001.tif]

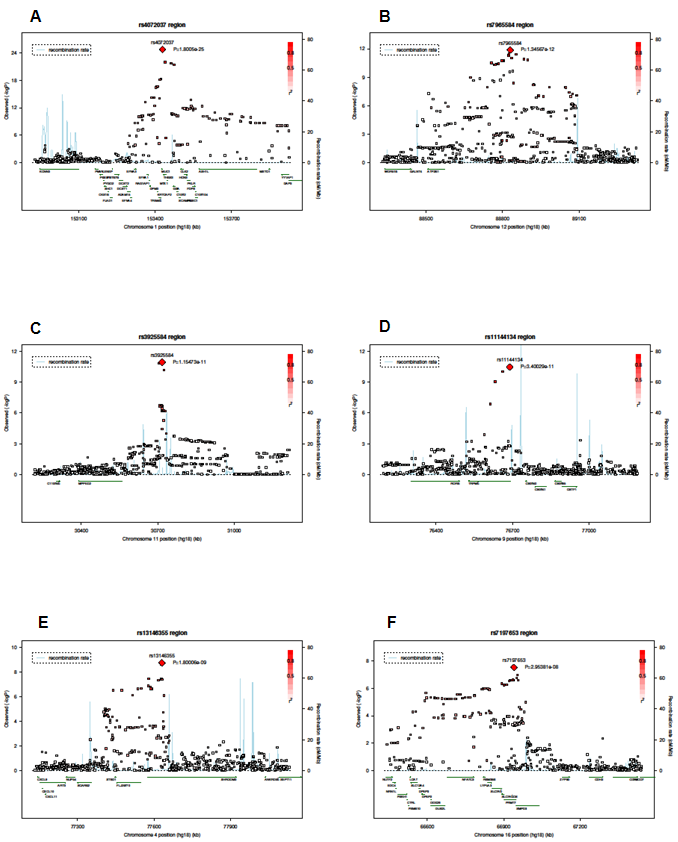

Supplement: Figure S2 — Regional association plots for SNPs and serum magnesium concentrations in 15,366 white participants from the CHARGE Consortium. Figures show −log10(p-values) by chromosomal position around the magnesium-associated regions along with any recombination hotspots in HapMap CEU. Genes that map within the regions are also noted on the plots. (A) SNPs in MUC1 region; (B) SNPs in ATP2B1 region; (C) SNPs in DCDC5 region; (D) SNPs in TRPM6 region; (E) SNPs in SHROOM3 region; (F) SNPs in PRMT7 region. (0.17 MB TIF) [file pgen.1001045.s002.tif]
