# Supplementary material for: Genome-Wide Association Studies of Serum Magnesium, Potassium, and Sodium Concentrations Identify Six Loci Influencing Serum Magnesium Levels
Source: PLoS Genet. 2010 Aug 5;6(8):e1001045. doi: 10.1371/journal.pgen.1001045 (PMC2916845; doi:10.1371/journal.pgen.1001045)
Supplement: Table S1 — Study-specific genotyping and imputation information for discovery and replication studies. (0.04 MB DOC) [file pgen.1001045.s003.doc]

**Table S1.** **Study-specific genotyping and imputation information for discovery and replication studies.**

| **Study** | **AGES** | **ARIC** | **CHS** | **FHS** | **KORA F3** | **KORA F4** | **RS** | **SHIP** |
| --- | --- | --- | --- | --- | --- | --- | --- | --- |
| Array | Illumina 370CNV | Affymetrix 6.0 | Illumina 370CNV | Affymetrix 500K;  Affymetrix 50K | Affymetrix 500K | Affymetrix 6.0 | Illumina 550K | Affymetrix 6.0 |
| Genotype calling | Illumina BeadStudio | Birdseed | Illumina BeadStudio | BRLMM | BRLMM | Birdseed 2 | Illumina BeadStudio | BirdseedV2 |
| Exclusion criteria for genotyped SNPs used for imputation | Call rate<97%; pHWE<10E-6; MAF<1%; Mishap P<10E-9; A/T and G/C SNPs; Mismatch between Illumina, dbSNP and/or HapMap position | Call rate<95%;  MAF<1%;  pHWE<10E-5 | Call rate<97%; heterozygotes=0; pHWE<10E-5; SNP not in HapMap | Call rate<95%;  pHWE<10E-6 | call rate < 90% HapMap SNPs only | call rate < 93% HapMap SNPs only | Call rate<90%; MAF<1%; pHWE<10E-6 | None |
| Imputation Software | MACH v.1.0.16 | MACH v.1.0.16 | BimBam | MACH v.1.0.15 | MACH v.1.0.9 | MACH v.1.0.15 | MACH v.1.0.15 | IMPUTEv0.5.0 |
| Number of SNPs used for imputation | 308,340 | 602,642 | 306,655 | 503,526 | 490,032 | 651,596 | 530,683 | 869,224 |
| Imputation Backbone (NCBI build) | HapMap CEU relesase 22 (build 36) | Phased CEU haploytpes, HapMap release 21 (build 35) | HapMap CEU release 21A, build 36 | Phased CEU haplotypes, HapMap release 22 (build 36) | HapMap release 21 (build 35) | HapMap release 22 (build 36) | Phased CEU haplotypes, HapMap release 22 (build 36) | HapMap CEU release 22 (build 36) |
| Exclusion criteria for imputed genotypes | None | None | Dosage variance ≤0.01 | None | None | None | None | None |
| Data management and statistical analysis | PLINK, R | ProbABEL, PLINK, R | R | R, lmekin function in Kinship package for continuous traits and gee function in GEE package for dichotomous traits | R, mach2qtl | R, mach2qtl | ProbABEL, R | R, Intersystems Caché, InforSense |

AGES, Age, Gene/Environment Susceptibility--Reykjavik Study; ARIC, The Atherosclerosis Risk in Communities Study; CHARGE, Cohorts for Heart and Aging Research in Genomic Epidemiology; CHS, The Cardiovascular Health Study; FHS, The Framingham Heart Study; HWE, Hardy-Weinberg equilibrium; KORA, Kooperative Gesundheitsforschung in der Region Augsburg; MAF, minor allele frequency; RS. The Rotterdam Study; SHIP, The Study of Health in Pomerania; SNP, single nucleotide polymorphism
