# Supplementary material for: Genome-Wide Association Studies of Serum Magnesium, Potassium, and Sodium Concentrations Identify Six Loci Influencing Serum Magnesium Levels
Source: PLoS Genet. 2010 Aug 5;6(8):e1001045. doi: 10.1371/journal.pgen.1001045 (PMC2916845; doi:10.1371/journal.pgen.1001045)
Supplement: Table S2 — SNP associations with serum sodium concentrations at p<10-5 in the CHARGE cohorts. (0.06 MB DOC) [file pgen.1001045.s004.doc]

**Table S2. SNP associations with serum sodium concentrations at p<10^-5^** in the CHARGE cohorts.

| **SNP** | **Coded Allele** | **Other Allele** | **P^*^** | **Chromosome** | **Location (bp)^†^** | **Closest Gene†** |
| --- | --- | --- | --- | --- | --- | --- |
| rs8007194 | a | g | 1.38E-06 | 14 | [69035425](http://www.ncbi.nlm.nih.gov/sites/nuccore/NC_000014.8?report=graph&v=69034925:69035925&content=5&m=69035425&mn=rs8007194&dispmax=1&currpage=1) | *RAD51L1* |
| rs2525503 | a | g | 4.55E-06 | 14 | [69028603](http://www.ncbi.nlm.nih.gov/sites/nuccore/NC_000014.8?report=graph&v=69028103:69029103&content=5&m=69028603&mn=rs2525503&dispmax=1&currpage=1) | *RAD51L1* |
| rs7153476 | t | g | 4.74E-06 | 14 | [69033230](http://www.ncbi.nlm.nih.gov/sites/nuccore/NC_000014.8?report=graph&v=69032730:69033730&content=5&m=69033230&mn=rs7153476&dispmax=1&currpage=1) | *RAD51L1* |
| rs12591546 | t | g | 4.04E-06 | 15 | [80754740](http://www.ncbi.nlm.nih.gov/sites/nuccore/NC_000015.9?report=graph&v=80754240:80755240&content=5&m=80754740&mn=rs12591546&dispmax=1&currpage=1) | *ARNT2* |
| rs2278709 | t | c | 4.32E-06 | 15 | [80743674](http://www.ncbi.nlm.nih.gov/sites/nuccore/NC_000015.9?report=graph&v=80743174:80744174&content=5&m=80743674&mn=rs2278709&dispmax=1&currpage=1) | *ARNT2* |
| rs17788150 | a | t | 4.47E-06 | 15 | [80743847](http://www.ncbi.nlm.nih.gov/sites/nuccore/NC_000015.9?report=graph&v=80743347:80744347&content=5&m=80743847&mn=rs17788150&dispmax=1&currpage=1) | *ARNT2* |
| rs3848175 | a | g | 5.28E-06 | 15 | [80747103](http://www.ncbi.nlm.nih.gov/sites/nuccore/NC_000015.9?report=graph&v=80746603:80747603&content=5&m=80747103&mn=rs3848175&dispmax=1&currpage=1) | *ARNT2* |
| rs12600036 | c | g | 4.12E-07 | 16 | [89230985](http://www.ncbi.nlm.nih.gov/sites/nuccore/NC_000016.9?report=graph&v=89230485:89231485&content=5&m=89230985&mn=rs12600036&dispmax=1&currpage=1) | *CDH15* |
| rs12599126 | t | c | 2.89E-06 | 16 | [89206483](http://www.ncbi.nlm.nih.gov/sites/nuccore/NC_000016.9?report=graph&v=89205983:89206983&content=5&m=89206483&mn=rs12599126&dispmax=1&currpage=1) | *LOC197322* |
| rs12325574 | a | g | 3.08E-06 | 16 | [89208334](http://www.ncbi.nlm.nih.gov/sites/nuccore/NC_000016.9?report=graph&v=89207834:89208834&content=5&m=89208334&mn=rs12325574&dispmax=1&currpage=1) | *LOC197322* |
| rs4352046 | t | c | 3.87E-06 | 16 | [89229088](http://www.ncbi.nlm.nih.gov/sites/nuccore/NC_000016.9?report=graph&v=89228588:89229588&content=5&m=89229088&mn=rs4352046&dispmax=1&currpage=1) | *LOC197322* |
| rs883941 | t | c | 4.00E-06 | 16 | [89226511](http://www.ncbi.nlm.nih.gov/sites/nuccore/NC_000016.9?report=graph&v=89226011:89227011&content=5&m=89226511&mn=rs883941&dispmax=1&currpage=1) | *LOC197322* |
| rs11640375 | a | g | 7.95E-06 | 16 | [89227817](http://www.ncbi.nlm.nih.gov/sites/nuccore/NC_000016.9?report=graph&v=89227317:89228317&content=5&m=89227817&mn=rs11640375&dispmax=1&currpage=1) | *LOC197322* |
| rs12598250 | a | g | 7.99E-06 | 16 | [89231051](http://www.ncbi.nlm.nih.gov/sites/nuccore/NC_000016.9?report=graph&v=89230551:89231551&content=5&m=89231051&mn=rs12598250&dispmax=1&currpage=1) | *CDH15* |
| rs6420563 | a | g | 6.72E-06 | 18 | [59586364](http://www.ncbi.nlm.nih.gov/sites/nuccore/NC_000018.9?report=graph&v=59585864:59586864&content=5&m=59586364&mn=rs6420563&dispmax=1&currpage=1) | *RNF152* |

^*^adjusted for age, sex, and center (if applicable); p-values are adjusted for genomic control; among subjects not using hypertension medications; †based on provisional NCBI genome build 37.1
