# Supplementary material for: Genome-Wide Association Studies of Serum Magnesium, Potassium, and Sodium Concentrations Identify Six Loci Influencing Serum Magnesium Levels
Source: PLoS Genet. 2010 Aug 5;6(8):e1001045. doi: 10.1371/journal.pgen.1001045 (PMC2916845; doi:10.1371/journal.pgen.1001045)
Supplement: Table S3 — SNP associations with serum potassium concentrations at p<10-5 in the CHARGE cohorts. (0.03 MB DOC) [file pgen.1001045.s005.doc]

**Table S3. SNP associations with serum potassium concentrations at p<10^-5^ in the CHARGE cohorts.**

| **SNP** | **Coded Allele** | **Other Allele** | **P^*^** | **Chromosome** | **Location (bp)^†^** | **Closest Gene†** |
| --- | --- | --- | --- | --- | --- | --- |
| rs1590118 | t | c | 1.66E-06 | 4 | [74534009](http://www.ncbi.nlm.nih.gov/sites/nuccore/NC_000004.11?report=graph&v=74533509:74534509&content=5&m=74534009&mn=rs1590118&dispmax=1&currpage=1) | *RASSF6* |
| rs13169121 | t | c | 7.43E-06 | 5 | [12759298](http://www.ncbi.nlm.nih.gov/sites/nuccore/NC_000005.8?report=graph&v=12758798:12759798&content=5&m=12759298&mn=rs13169121&dispmax=1&currpage=1) | *CTNND2* |
| rs6471999 | a | g | 4.99E-06 | 8 | [63140630](http://www.ncbi.nlm.nih.gov/sites/nuccore/NC_000008.10?report=graph&v=63140130:63141130&content=5&m=63140630&mn=rs2882740&dispmax=1&currpage=1) |  |
| rs945387 | t | c | 3.34E-06 | 9 | [139693992](http://www.ncbi.nlm.nih.gov/sites/nuccore/NC_000009.11?report=graph&v=139693492:139694492&content=5&m=139693992&mn=rs945387&dispmax=1&currpage=1) | *KIAA1984* |
| rs878396 | a | g | 6.72E-06 | 18 | [55811058](http://www.ncbi.nlm.nih.gov/sites/nuccore/NC_000018.9?report=graph&v=55810558:55811558&content=5&m=55811058&mn=rs878396&dispmax=1&currpage=1) | *NEDD4L* |
| rs4149589 | t | c | 2.24E-06 | 18 | [55815017](http://www.ncbi.nlm.nih.gov/sites/nuccore/NC_000018.9?report=graph&v=55814517:55815517&content=5&m=55815017&mn=rs4149589&dispmax=1&currpage=1) | *NEDD4L* |

^*^adjusted for age, sex, and center (if applicable); p-values are adjusted for genomic control; among subjects not using hypertension medications; †based on provisional NCBI genome build 37.1
