# Supplementary material for: Genome-Wide Association Studies of Serum Magnesium, Potassium, and Sodium Concentrations Identify Six Loci Influencing Serum Magnesium Levels
Source: PLoS Genet. 2010 Aug 5;6(8):e1001045. doi: 10.1371/journal.pgen.1001045 (PMC2916845; doi:10.1371/journal.pgen.1001045)
Supplement: Table S4 — Study-specific associations for magnesium levels and the lead regional magnesium genome-wide association study hits in the discovery cohorts. (0.05 MB DOC) [file pgen.1001045.s006.doc]

**Table S4.** **Study-specific associations for magnesium levels and the lead regional magnesium genome-wide association study hits in the discovery cohorts.**

|  |  |  | **ARIC (N=8,122)** | | | **FHS (N=2,866)** | | | **RS (N=4,378)** | | | **Discovery Sample (N=15,366)** | | |
| --- | --- | --- | --- | --- | --- | --- | --- | --- | --- | --- | --- | --- | --- | --- |
| **SNP** | **CHR** | **Closest Gene** | **Beta (mmol/L)** | **SE** | **P** | **Beta (mmol/L)** | **SE** | **P** | **Beta (mmol/L)** | **SE** | **P** | **Beta (mmol/L)** | **SE** | **P** |
| *SNPs with genome-wide significant association after discovery (p<5x10^-8^)* | | | | | | | | | | | | | | |
| rs4072037 | 1 | *MUC1* | -0.011 | 0.001 | 3.50E-21 | -0.007 | 0.003 | 6.77E-03 | -0.008 | 0.002 | 2.95E-05 | -0.010 | 9.00E-04 | 1.80E-25 |
| rs13146355 | 4 | *SHROOM3* | -0.006 | 0.001 | 2.95E-07 | -0.007 | 0.002 | 1.65E-03 | -0.003 | 0.002 | 9.68E-02 | -0.006 | 9.00E-04 | 1.80E-09 |
| rs11144134 | 9 | *TRPM6* | -0.013 | 0.002 | 5.65E-09 | -0.012 | 0.004 | 1.99E-03 | -0.007 | 0.003 | 5.47E-02 | -0.011 | 0.002 | 3.40E-11 |
| rs3925584 | 11 | *DCDC5* | -0.008 | 0.001 | 1.32E-10 | -0.007 | 0.002 | 6.31E-04 | -0.002 | 0.002 | 3.45E-01 | -0.006 | 9.00E-04 | 1.15E-11 |
| rs7965584 | 12 | *ATP2B1* | -0.008 | 0.001 | 1.14E-09 | -0.004 | 0.002 | 1.40E-01 | -0.008 | 0.002 | 1.66E-04 | -0.007 | 0.001 | 1.35E-12 |
| rs7197653 | 16 | *PRMT7* | -0.009 | 0.002 | 7.00E-07 | -0.008 | 0.003 | 2.39E-02 | -0.005 | 0.003 | 9.18E-02 | -0.008 | 0.001 | 2.95E-08 |
| *SNPs with suggestive association after discovery (p<4x10^-7^)* | | | | | | | | | | | | | | |
| rs2592394 | 2 | *HOXD9* | -0.005 | 0.001 | 5.83E-04 | -0.008 | 0.002 | 5.43E-04 | -0.005 | 0.002 | 2.81E-02 | -0.005 | 0.001 | 3.20E-07 |
| rs448378 | 3 | *MDS1* | -0.006 | 0.001 | 5.94E-07 | -0.003 | 0.002 | 2.29E-01 | -0.003 | 0.002 | 8.60E-02 | -0.005 | 9.00E-04 | 2.64E-07 |
| rs4561213 | 11 | *LUZP2* | -0.005 | 0.001 | 1.71E-04 | 0.004 | 0.002 | 4.37E-02 | -0.006 | 0.002 | 1.00E-03 | -0.005 | 9.00E-04 | 1.35E-07 |

ARIC, The Atherosclerosis Risk in Communities Study; CHR, chromosome; FHS, The Framingham Heart Study; RS, The Rotterdam Study; SE, standard error; SNP, single nucleotide polymorphism.
