# Supplementary material for: Genome-Wide Association Studies of Serum Magnesium, Potassium, and Sodium Concentrations Identify Six Loci Influencing Serum Magnesium Levels
Source: PLoS Genet. 2010 Aug 5;6(8):e1001045. doi: 10.1371/journal.pgen.1001045 (PMC2916845; doi:10.1371/journal.pgen.1001045)
Supplement: Table S5 — SNP association with serum magnesium concentrations at p<10-6 in the CHARGE cohorts. (0.46 MB DOC) [file pgen.1001045.s007.doc]

**Table S5. SNP associations with serum magnesium concentrations at p<10^-6^ in the CHARGE cohorts.**

| **SNP** | **Coded Allele** | **Other Allele** | **P^*^** | **Chromosome** | **Location (bp)^†^** | **Closest Gene†** |
| --- | --- | --- | --- | --- | --- | --- |
| rs1462855 | t | c | 2.27E-09 | 1 | 155053719 | *EFNA3* |
| rs7534162 | a | g | 2.06E-09 | 1 | 155065159 | *EFNA3* |
| rs10908455 | t | c | 7.98E-08 | 1 | 155067283 | *EFNA3* |
| rs7365544 | a | c | 9.61E-11 | 1 | 155068748 | *EFNA3* |
| rs6427158 | t | g | 8.85E-11 | 1 | 155074903 | *EFNA3* |
| rs11264314 | c | g | 8.37E-11 | 1 | 155078423 | *EFNA3* |
| rs7367207 | t | c | 6.30E-08 | 1 | 155079477 | *EFNA3* |
| rs7368345 | a | g | 6.78E-11 | 1 | 155080090 | *EFNA3* |
| rs6693477 | t | c | 6.35E-11 | 1 | 155081940 | *EFNA1* |
| rs7548955 | t | c | 6.33E-11 | 1 | 155082298 | *EFNA1* |
| rs7367897 | a | t | 4.45E-10 | 1 | 155083289 | *EFNA1* |
| rs11264318 | a | t | 6.31E-11 | 1 | 155083717 | *EFNA1* |
| rs11264319 | t | c | 6.32E-11 | 1 | 155083942 | *EFNA1* |
| rs10908456 | a | g | 5.72E-08 | 1 | 155086148 | *EFNA1* |
| rs11264329 | a | g | 6.14E-14 | 1 | 155095158 | *EFNA1* |
| rs4255378 | a | t | 2.22E-11 | 1 | 155106227 | *EFNA1* |
| rs9297 | a | g | 3.32E-11 | 1 | 155106550 | *EFNA1* |
| rs4246529 | t | c | 4.24E-11 | 1 | 155115260 | *DPM3* |
| rs12025371 | a | t | 5.95E-15 | 1 | 155119327 | *ADAM15* |
| rs10157801 | a | g | 3.63E-11 | 1 | 155120012 | *DPM3* |
| rs6427190 | a | c | 2.51E-08 | 1 | 155124772 | *KRTCAP2* |
| rs4971052 | t | c | 4.00E-10 | 1 | 155126018 | *KRTCAP2* |
| rs10908458 | t | c | 2.64E-11 | 1 | 155126948 | *DPM3* |
| rs4971079 | a | g | 5.43E-15 | 1 | 155130391 | *KRTCAP2* |
| rs4276913 | a | g | 3.43E-13 | 1 | 155131673 | *KRTCAP2* |
| rs4460629 | t | c | 3.93E-17 | 1 | 155135335 | *KRTCAP2* |
| rs11264339 | t | c | 2.50E-17 | 1 | 155140648 | *KRTCAP2* |
| rs4971088 | a | t | 1.94E-17 | 1 | 155142883 | *KRTCAP2* |
| rs3814316 | a | g | 8.35E-15 | 1 | 155149718 | *TRIM46* |
| rs11264341 | t | c | 7.72E-19 | 1 | 155151493 | *TRIM46* |
| rs9426886 | a | t | 7.40E-19 | 1 | 155151754 | *TRIM46* |
| rs4971100 | a | g | 4.79E-19 | 1 | 155155731 | *TRIM46* |
| rs4072037 | t | c | 1.80E-25 | 1 | 155162067 | *MUC1* |
| rs2066981 | a | g | 9.94E-23 | 1 | 155172379 | *THBS3* |
| rs2049805 | t | c | 1.72E-22 | 1 | 155194980 | *GBAP* |
| rs2990245 | t | c | 1.78E-22 | 1 | 155197462 | *GBA* |
| rs12407919 | c | g | 1.93E-11 | 1 | 155199315 | *GBA* |
| rs3768566 | t | c | 1.70E-11 | 1 | 155201064 | *GBA* |
| rs1045253 | a | g | 2.88E-11 | 1 | 155201235 | *GBA* |
| rs9628662 | t | g | 2.51E-11 | 1 | 155206341 | *GBA* |
| rs364897 | t | c | 4.09E-22 | 1 | 155208006 | *GBA* |
| rs11264345 | a | t | 3.20E-11 | 1 | 155213124 | *GBA* |
| rs734073 | t | c | 5.85E-11 | 1 | 155218365 | *C1orf2* |
| rs2242577 | a | c | 6.58E-11 | 1 | 155224301 | *FAM189B* |
| rs2236863 | a | g | 1.22E-12 | 1 | 155241361 | *CLK2* |
| rs7549276 | a | g | 1.35E-12 | 1 | 155248575 | *HCN3* |
| rs7520184 | a | g | 1.44E-12 | 1 | 155253583 | *HCN3* |
| rs11264352 | t | c | 1.46E-12 | 1 | 155255837 | *HCN3* |
| rs11264355 | c | g | 2.09E-12 | 1 | 155257492 | *HCN3* |
| rs932972 | a | g | 2.10E-12 | 1 | 155260096 | *PKLR* |
| rs1052176 | t | g | 2.11E-12 | 1 | 155260383 | *PKLR* |
| rs4620533 | c | g | 1.95E-12 | 1 | 155262613 | *PKLR* |
| rs3020781 | a | g | 1.70E-12 | 1 | 155269776 | *PKLR* |
| rs4971072 | a | g | 1.99E-14 | 1 | 155273869 | *FDPS* |
| rs12032720 | c | g | 4.29E-13 | 1 | 155274960 | *FDPS* |
| rs2297480 | t | g | 9.15E-13 | 1 | 155279482 | *FDPS* |
| rs11264359 | a | g | 3.48E-12 | 1 | 155282829 | *FDPS* |
| rs11264361 | t | g | 1.74E-12 | 1 | 155289545 | *FDPS* |
| rs11264363 | c | g | 4.66E-13 | 1 | 155318308 | *ASH1L* |
| rs12239114 | a | g | 6.69E-11 | 1 | 155322142 | *ASH1L* |
| rs11264367 | t | c | 7.79E-11 | 1 | 155337338 | *ASH1L* |
| rs5005770 | a | g | 2.11E-10 | 1 | 155345043 | *ASH1L* |
| rs11264369 | t | c | 5.09E-10 | 1 | 155359830 | *ASH1L* |
| rs7556102 | a | c | 1.05E-11 | 1 | 155368203 | *ASH1L* |
| rs1886905 | t | c | 5.33E-10 | 1 | 155372672 | *ASH1L* |
| rs10908465 | t | c | 2.46E-10 | 1 | 155389688 | *ASH1L* |
| rs12041534 | t | c | 2.54E-10 | 1 | 155407096 | *ASH1L* |
| rs11264371 | t | c | 5.53E-09 | 1 | 155412037 | *ASH1L* |
| rs11264372 | a | g | 5.57E-09 | 1 | 155412930 | *ASH1L* |
| rs1325908 | a | c | 5.41E-10 | 1 | 155413304 | *ASH1L* |
| rs10796943 | t | c | 4.13E-10 | 1 | 155414729 | *ASH1L* |
| rs12730906 | t | c | 3.58E-07 | 1 | 155414768 | *ASH1L* |
| rs11264375 | t | c | 3.37E-10 | 1 | 155424065 | *ASH1L* |
| rs6684889 | a | t | 2.52E-11 | 1 | 155427062 | *ASH1L* |
| rs12724079 | t | c | 2.65E-11 | 1 | 155433942 | *ASH1L* |
| rs12079134 | c | g | 9.20E-10 | 1 | 155466784 | *ASH1L* |
| rs10908469 | a | c | 1.06E-09 | 1 | 155468732 | *ASH1L* |
| rs1360554 | t | c | 8.81E-11 | 1 | 155473356 | *ASH1L* |
| rs6688636 | t | c | 1.21E-10 | 1 | 155478897 | *ASH1L* |
| rs12746592 | a | g | 1.53E-09 | 1 | 155505967 | *ASH1L* |
| rs6696888 | a | g | 1.31E-10 | 1 | 155508882 | *ASH1L* |
| rs10752611 | t | c | 1.92E-09 | 1 | 155509107 | *ASH1L* |
| rs10908470 | t | g | 1.99E-09 | 1 | 155527013 | *ASH1L* |
| rs1556488 | t | g | 2.02E-09 | 1 | 155540660 | *ASH1L* |
| rs10908471 | a | g | 2.02E-09 | 1 | 155542942 | *ASH1L* |
| rs3892792 | t | g | 2.02E-09 | 1 | 155558665 | *MSTO1* |
| rs11264387 | a | c | 2.04E-09 | 1 | 155563152 | *MSTO1* |
| rs11264397 | c | g | 8.40E-09 | 1 | 155611485 | *YY1AP1* |
| rs12039893 | a | c | 1.45E-07 | 1 | 155614815 | *YY1AP1* |
| rs2048431 | a | g | 8.50E-09 | 1 | 155618630 | *YY1AP1* |
| rs2175391 | t | g | 8.84E-09 | 1 | 155625602 | *YY1AP1* |
| rs3738590 | a | g | 8.20E-09 | 1 | 155630752 | *YY1AP1* |
| rs10908479 | t | c | 8.33E-09 | 1 | 155643403 | *YY1AP1* |
| rs475550 | a | t | 8.40E-09 | 1 | 155652081 | *YY1AP1* |
| rs678157 | t | c | 1.12E-07 | 1 | 155658341 | *DAP4* |
| rs12744132 | t | c | 1.24E-07 | 1 | 155663326 | *DAP3* |
| rs670876 | t | g | 1.25E-07 | 1 | 155671678 | *DAP3* |
| rs821551 | a | c | 1.76E-08 | 1 | 155688580 | *DAP5* |
| rs10908481 | t | g | 6.16E-07 | 1 | 155713650 | *DAP3* |
| rs394851 | t | g | 3.18E-08 | 1 | 155719467 | *GON4L* |
| rs7541060 | a | t | 5.22E-08 | 1 | 155728088 | *GON4L* |
| rs2297775 | t | c | 6.96E-07 | 1 | 155735012 | *GON4L* |
| rs708611 | a | c | 3.38E-08 | 1 | 155743310 | *GON4L* |
| rs822490 | t | c | 8.71E-08 | 1 | 155822971 | *GON4L* |
| rs2592394 | a | g | 3.20E-07 | 2 | 176991779 | *HOXD9* |
| rs1290790 | a | t | 8.31E-07 | 3 | 169091569 | *MDS1* |
| rs1290784 | t | c | 4.40E-07 | 3 | 169096900 | *MDS1* |
| rs1290786 | t | c | 7.70E-07 | 3 | 169097381 | *MDS1* |
| rs988397 | t | c | 7.70E-07 | 3 | 169098791 | *MDS1* |
| rs988398 | t | c | 5.46E-07 | 3 | 169098861 | *MDS1* |
| rs223102 | t | c | 2.72E-07 | 3 | 169100755 | *MDS1* |
| rs419076 | t | c | 2.69E-07 | 3 | 169100886 | *MDS1* |
| rs448378 | a | g | 2.64E-07 | 3 | 169100899 | *MDS1* |
| rs6779380 | t | c | 3.23E-07 | 3 | 169111915 | *MDS1* |
| rs2421647 | a | c | 3.30E-07 | 3 | 169113110 | *MDS1* |
| rs9290366 | a | g | 3.39E-07 | 3 | 169117005 | *MDS1* |
| rs4955647 | a | t | 9.14E-07 | 3 | 169121931 | *MDS1* |
| rs1918966 | a | g | 5.42E-07 | 3 | 169128274 | *MDS1* |
| rs7626980 | a | g | 8.88E-07 | 3 | 169155318 | *MDS1* |
| rs1918974 | t | c | 9.57E-07 | 3 | 169165888 | *MDS1* |
| rs4955658 | t | g | 9.59E-07 | 3 | 169171347 | *MDS1* |
| rs12506745 | t | g | 3.05E-07 | 4 | 77220837 | *STBD1* |
| rs907446 | t | c | 3.12E-07 | 4 | 77254804 | *CCDC158* |
| rs1876535 | t | c | 7.44E-07 | 4 | 77296600 | *CCDC158* |
| rs6857452 | t | c | 2.34E-07 | 4 | 77317124 | *CCDC158* |
| rs4272041 | a | g | 2.13E-07 | 4 | 77330682 | *SHROOM3* |
| rs10006043 | t | c | 1.31E-07 | 4 | 77357747 | *SHROOM3* |
| rs10023335 | t | c | 3.68E-08 | 4 | 77358987 | *SHROOM3* |
| rs9992101 | a | g | 3.46E-08 | 4 | 77360431 | *SHROOM3* |
| rs17253722 | a | g | 1.69E-07 | 4 | 77367287 | *SHROOM3* |
| rs1398016 | a | g | 1.66E-07 | 4 | 77367688 | *SHROOM3* |
| rs17319721 | a | g | 2.36E-07 | 4 | 77368847 | *SHROOM3* |
| rs1398018 | t | c | 2.29E-07 | 4 | 77372923 | *SHROOM3* |
| rs10032549 | a | g | 4.59E-08 | 4 | 77398015 | *SHROOM3* |
| rs7654754 | a | g | 3.70E-08 | 4 | 77409795 | *SHROOM3* |
| rs7656186 | t | c | 3.75E-08 | 4 | 77409818 | *SHROOM3* |
| rs7654978 | t | g | 3.80E-08 | 4 | 77409945 | *SHROOM3* |
| rs4859682 | a | c | 2.39E-09 | 4 | 77410318 | *SHROOM3* |
| rs13146355 | a | g | 1.80E-09 | 4 | 77412140 | *SHROOM3* |
| rs4318673 | a | t | 4.02E-08 | 4 | 77412338 | *SHROOM3* |
| rs7674982 | a | g | 4.07E-08 | 4 | 77412997 | *SHROOM3* |
| rs7675258 | a | g | 4.10E-08 | 4 | 77413179 | *SHROOM3* |
| rs1986734 | t | c | 8.41E-07 | 4 | 77420784 | *SHROOM3* |
| rs10109512 | t | c | 7.45E-07 | 8 | 23121059 | *R3HCC1* |
| rs12380414 | t | c | 1.34E-07 | 9 | 77422605 | *TRPM6* |
| rs11144105 | a | t | 8.72E-10 | 9 | 77442370 | *TRPM6* |
| rs12378991 | a | g | 9.37E-11 | 9 | 77472066 | *TRPM6* |
| rs11144134 | t | c | 3.40E-11 | 9 | 77499796 | *TRPM6* |
| rs3740393 | c | g | 8.58E-07 | 10 | 104636655 | *AS3MT* |
| rs4561213 | t | g | 1.35E-07 | 11 | 24678819 | *LUZP2* |
| rs4465366 | t | c | 3.69E-07 | 11 | 24683117 | *LUZP2* |
| rs963837 | t | c | 1.52E-11 | 11 | 30749090 | *DCDC5* |
| rs795237 | t | c | 1.74E-07 | 11 | 30751010 | *DCDC5* |
| rs685270 | t | c | 2.40E-07 | 11 | 30751955 | *DCDC5* |
| rs683877 | t | c | 1.78E-07 | 11 | 30752278 | *DCDC5* |
| rs583143 | a | c | 1.74E-07 | 11 | 30755064 | *DCDC5* |
| rs628029 | t | g | 1.73E-07 | 11 | 30758164 | *DCDC5* |
| rs628093 | a | g | 5.99E-07 | 11 | 30758215 | *DCDC5* |
| rs628941 | a | g | 6.04E-07 | 11 | 30758377 | *DCDC5* |
| rs686864 | t | c | 2.00E-07 | 11 | 30759583 | *DCDC5* |
| rs3925584 | t | c | 1.15E-11 | 11 | 30760335 | *DCDC5* |
| rs1232066 | t | g | 3.83E-07 | 11 | 30760968 | *DCDC5* |
| rs582740 | t | g | 1.97E-07 | 11 | 30761557 | *DCDC5* |
| rs629681 | t | c | 6.68E-07 | 11 | 30761727 | *DCDC5* |
| rs627920 | a | t | 6.49E-07 | 11 | 30762105 | *DCDC5* |
| rs613950 | t | c | 6.16E-07 | 11 | 30762936 | *DCDC5* |
| rs599448 | a | g | 5.95E-07 | 11 | 30762964 | *DCDC5* |
| rs613329 | t | c | 5.78E-07 | 11 | 30763792 | *DCDC5* |
| rs598744 | a | g | 5.71E-07 | 11 | 30764079 | *DCDC5* |
| rs627257 | a | g | 5.69E-07 | 11 | 30764611 | *DCDC5* |
| rs628568 | a | g | 5.65E-07 | 11 | 30764890 | *DCDC5* |
| rs677823 | a | t | 5.57E-07 | 11 | 30765841 | *DCDC5* |
| rs661296 | t | c | 5.56E-07 | 11 | 30767193 | *DCDC5* |
| rs684695 | a | g | 5.75E-07 | 11 | 30768229 | *DCDC5* |
| rs1232070 | t | c | 6.00E-07 | 11 | 30768620 | *DCDC5* |
| rs10767873 | t | c | 6.38E-11 | 11 | 30768678 | *DCDC5* |
| rs795230 | t | c | 6.72E-07 | 11 | 30774525 | *DCDC5* |
| rs10506975 | t | c | 5.24E-08 | 12 | 89986523 | *ATP2B1* |
| rs17017109 | t | g | 2.03E-07 | 12 | 90004107 | *ATP2B1* |
| rs10858915 | a | g | 5.25E-07 | 12 | 90051547 | *ATP2B1* |
| rs7399069 | t | g | 2.94E-07 | 12 | 90109321 | *NA* |
| rs4842684 | t | c | 4.23E-07 | 12 | 90143162 | *ATP2B1* |
| rs11105401 | a | g | 1.02E-09 | 12 | 90150382 | *ATP2B1* |
| rs12579003 | t | c | 2.31E-09 | 12 | 90161860 | *ATP2B1* |
| rs12578803 | a | g | 1.43E-09 | 12 | 90186086 | *ATP2B1* |
| rs7136711 | a | g | 1.13E-08 | 12 | 90215621 | *ATP2B1* |
| rs11105429 | t | c | 3.02E-11 | 12 | 90231778 | *ATP2B1* |
| rs12828794 | a | c | 2.16E-09 | 12 | 90236293 | *ATP2B1* |
| rs12828942 | t | c | 2.27E-09 | 12 | 90236325 | *ATP2B1* |
| rs9788161 | t | c | 2.82E-09 | 12 | 90243047 | *ATP2B1* |
| rs10506978 | a | g | 4.80E-11 | 12 | 90244121 | *ATP2B1* |
| rs7294375 | t | g | 4.77E-11 | 12 | 90247390 | *ATP2B1* |
| rs11105439 | t | c | 4.11E-11 | 12 | 90251760 | *ATP2B1* |
| rs11105442 | c | g | 3.62E-11 | 12 | 90260348 | *ATP2B1* |
| rs11105443 | a | t | 3.60E-11 | 12 | 90260446 | *ATP2B1* |
| rs12424558 | c | g | 1.01E-07 | 12 | 90266133 | *ATP2B1* |
| rs17192310 | a | g | 2.02E-09 | 12 | 90267134 | *ATP2B1* |
| rs7975788 | t | g | 1.82E-11 | 12 | 90273927 | *ATP2B1* |
| rs7960337 | t | c | 1.77E-11 | 12 | 90277578 | *ATP2B1* |
| rs11105452 | a | c | 1.75E-11 | 12 | 90279165 | *ATP2B1* |
| rs12811730 | c | g | 6.27E-10 | 12 | 90281515 | *ATP2B1* |
| rs10777209 | t | c | 5.19E-12 | 12 | 90299647 | *ATP2B1* |
| rs10858938 | t | g | 3.60E-12 | 12 | 90301679 | *ATP2B1* |
| rs12823723 | a | g | 2.72E-10 | 12 | 90304545 | *ATP2B1* |
| rs7965584 | a | g | 1.35E-12 | 12 | 90305779 | *ATP2B1* |
| rs11105462 | a | g | 9.15E-12 | 12 | 90311971 | *ATP2B1* |
| rs4132405 | a | g | 2.26E-10 | 12 | 90315195 | *ATP2B1* |
| rs7314270 | t | c | 7.46E-07 | 12 | 90317290 | *ATP2B1* |
| rs12229946 | t | g | 4.25E-09 | 12 | 90319258 | *ATP2B1* |
| rs11105468 | a | t | 3.75E-12 | 12 | 90328833 | *ATP2B1* |
| rs4503596 | a | g | 1.23E-11 | 12 | 90360364 | *ATP2B1* |
| rs10858941 | t | c | 1.52E-11 | 12 | 90375142 | *ATP2B1* |
| rs7964216 | t | c | 6.22E-09 | 12 | 90396480 | *ATP2B1* |
| rs10777218 | t | c | 6.00E-09 | 12 | 90398262 | *ATP2B1* |
| rs11105483 | t | c | 6.26E-09 | 12 | 90399351 | *ATP2B1* |
| rs6538216 | a | t | 1.50E-07 | 12 | 90408211 | *ATP2B1* |
| rs10777219 | a | c | 9.21E-08 | 12 | 90416357 | *ATP2B1* |
| rs10858943 | a | g | 9.37E-08 | 12 | 90418122 | *ATP2B1* |
| rs7975953 | t | c | 1.05E-07 | 12 | 90436082 | *ATP2B1* |
| rs7980592 | a | g | 1.11E-08 | 12 | 90460256 | *ATP2B1* |
| rs10858959 | t | c | 1.12E-08 | 12 | 90522149 | *ATP2B1* |
| rs10858960 | a | g | 1.20E-08 | 12 | 90523703 | *ATP2B1* |
| rs7313782 | a | g | 4.22E-08 | 12 | 90537270 | *ATP2B1* |
| rs10777230 | a | g | 1.05E-07 | 12 | 90543507 | *ATP2B1* |
| rs10858962 | t | c | 7.19E-07 | 12 | 90545220 | *ATP2B1* |
| rs10858963 | t | c | 7.32E-07 | 12 | 90546388 | *ATP2B1* |
| rs1438982 | a | g | 7.37E-07 | 12 | 90546799 | *ATP2B1* |
| rs1371078 | a | t | 7.40E-07 | 12 | 90546974 | *ATP2B1* |
| rs7134495 | a | g | 5.95E-07 | 12 | 90549231 | *ATP2B1* |
| rs11105520 | t | g | 5.96E-08 | 12 | 90550853 | *ATP2B1* |
| rs2408224 | a | g | 8.15E-07 | 12 | 90551579 | *ATP2B1* |
| rs2099667 | t | c | 8.88E-07 | 12 | 90554814 | *ATP2B1* |
| rs2083260 | a | g | 9.25E-07 | 12 | 90554983 | *ATP2B1* |
| rs2579090 | a | g | 9.41E-07 | 12 | 90555885 | *ATP2B1* |
| rs10858966 | c | g | 8.19E-08 | 12 | 90567026 | *ATP2B1* |
| rs1438998 | a | t | 8.03E-08 | 12 | 90567732 | *ATP2B1* |
| rs11350 | t | c | 4.38E-07 | 16 | 68335392 | *SLC7A6OS* |
| rs8063446 | a | c | 5.63E-07 | 16 | 68344363 | *SLC7A6OS* |
| rs7189887 | t | c | 5.30E-07 | 16 | 68353915 | *PRMT7* |
| rs7190070 | t | c | 5.31E-07 | 16 | 68353983 | *PRMT7* |
| rs7500163 | c | g | 2.98E-07 | 16 | 68364358 | *PRMT7* |
| rs3785113 | t | c | 2.50E-07 | 16 | 68369213 | *PRMT7* |
| rs3785116 | a | g | 2.48E-07 | 16 | 68372730 | *PRMT7* |
| rs16957831 | t | c | 2.49E-07 | 16 | 68378341 | *PRMT7* |
| rs8058517 | t | c | 2.42E-07 | 16 | 68379860 | *PRMT7* |
| rs7190134 | a | g | 2.56E-07 | 16 | 68381516 | *PRMT7* |
| rs7197653 | c | g | 2.95E-08 | 16 | 68383047 | *PRMT7* |
| rs12599876 | t | c | 1.80E-07 | 16 | 68393405 | *SMPD3* |
| rs2279538 | c | g | 1.09E-07 | 16 | 68397106 | *SMPD3* |
| rs3785127 | c | g | 2.50E-07 | 16 | 68399590 | *SMPD3* |

^*^adjusted for age, sex, and center (if applicable); p-values are adjusted for genomic control; ^†^based on provisional NCBI genome build 37.1
