# Supplementary material for: Genome-Wide Association Studies of Serum Magnesium, Potassium, and Sodium Concentrations Identify Six Loci Influencing Serum Magnesium Levels
Source: PLoS Genet. 2010 Aug 5;6(8):e1001045. doi: 10.1371/journal.pgen.1001045 (PMC2916845; doi:10.1371/journal.pgen.1001045)
Supplement: Table S6 — Study-specific associations for magnesium levels and the lead regional magnesium genome-wide association study hits in the replication cohorts. (0.05 MB DOC) [file pgen.1001045.s008.doc]

**Table S6.** **Study-specific associations for magnesium levels and the lead regional magnesium genome-wide association study hits in the replication cohorts.**

|  |  |  | **ARIC (N=948)** | | | **KORA F3 (N=1,641)** | | | **KORA F4(N=1,809)** | | | **SHIP (N=4,065)** | | | **Replication Sample (N=8,463)** | | | |
| --- | --- | --- | --- | --- | --- | --- | --- | --- | --- | --- | --- | --- | --- | --- | --- | --- | --- | --- |
| **SNP** | **CHR** | **Closest Gene** | **Beta (mmol/L)** | **SE** | **P** | **Beta (mmol/L)** | **SE** | **P** | **Beta (mmol/L)** | **SE** | **P** | **Beta (mmol/L)** | **SE** | **P** | **Beta (mmol/L)** | | **SE** | **P** |
| *SNPs with genome-wide significant association after discovery (p<5x10^-8^)* | | | | | | | | | | | | | | | | | | |
| rs4072037 | 1 | *MUC1* | -0.006 | 0.004 | 9.17E-02 | -0.014 | 0.003 | 1.27E-06 | -0.015 | 0.002 | 5.91E-12 | -0.001 | 0.002 | 4.88E-01 | | -0.009 | 0.001 | 2.12E-12 |
| rs13146355 | 4 | *SHROOM3* | -0.003 | 0.003 | 3.15E-01 | -0.010 | 0.003 | 1.28E-04 | -0.003 | 0.002 | 1.36E-01 | -0.003 | 0.002 | 1.01E-01 | | -0.005 | 0.001 | 8.44E-05 |
| rs11144134 | 9 | *TRPM6* | -0.016 | 0.007 | 1.57E-02 | -0.011 | 0.008 | 1.52E-01 | -0.008 | 0.005 | 7.52E-02 | -0.010 | 0.004 | 1.43E-02 | | -0.010 | 0.003 | 6.41E-05 |
| rs3925584 | 11 | *DCDC5* | -0.007 | 0.003 | 3.36E-02 | -0.006 | 0.002 | 2.33E-02 | -0.008 | 0.002 | 3.54E-04 | -0.002 | 0.002 | 2.91E-01 | | -0.005 | 0.001 | 9.60E-06 |
| rs7965584 | 12 | *ATP2B1* | -0.007 | 0.004 | 7.43E-02 | -0.004 | 0.003 | 1.40E-01 | -0.007 | 0.002 | 4.40E-03 | -0.005 | 0.002 | 1.39E-02 | | -0.006 | 0.001 | 1.44E-05 |
| rs7197653 | 16 | *PRMT7* | -4.33E-04 | 0.005 | 9.30E-01 | 0.002 | 0.004 | 6.05E-01 | 0.001 | 0.003 | 6.71E-01 | -0.005 | 0.003 | 9.65E-02 | | -0.001 | 0.002 | 5.92E-01 |
| *SNPs with suggestive association after discovery (p<4x10^-7^)* | | | | | | | | | | | | | | | | | | |
| rs2592394 | 2 | *HOXD9* | -0.009 | 0.004 | 2.04E-02 | -4.49E-04 | 0.003 | 8.82E-01 | -0.003 | 0.002 | 2.88E-01 | -2.73E-04 | 0.002 | 9.04E-01 | | -0.002 | 0.001 | 1.18E-01 |
| rs448378 | 3 | *MDS1* | 0.001 | 0.003 | 6.47E-01 | -0.006 | 0.002 | 1.13E-02 | -0.002 | 0.002 | 2.90E-01 | -0.003 | 0.002 | 9.29E-02 | | -0.003 | 0.001 | 9.29E-03 |
| rs4561213 | 11 | *LUZP2* | -0.006 | 0.003 | 9.77E-02 | -0.004 | 0.003 | 1.01E-01 | 0.001 | 0.002 | 7.15E-01 | -0.001 | 0.002 | 5.13E-01 | | -0.002 | 0.001 | 1.28E-01 |

ARIC, The Atherosclerosis Risk in Communities Study; CHR, chromosome; KORA, Kooperative Gesundheitsforschung in der Region Augsburg; SE, standard error; SHIP, The Study of Health in Pomerania; SNP, single nucleotide polymorphism.
