# Supplementary material for: Genome-Wide Association Studies of Serum Magnesium, Potassium, and Sodium Concentrations Identify Six Loci Influencing Serum Magnesium Levels
Source: PLoS Genet. 2010 Aug 5;6(8):e1001045. doi: 10.1371/journal.pgen.1001045 (PMC2916845; doi:10.1371/journal.pgen.1001045)
Supplement: Table S7 — Imputation quality for SNPs with significant and suggestive association with serum magnesium concentrations. (0.04 MB DOC) [file pgen.1001045.s009.doc]

**Table S7. Imputation quality for SNPs with significant and suggestive association with serum magnesium concentrations.**

|  | **CHARGE Cohorts** | | | | | **Replication Samples** | | | |
| --- | --- | --- | --- | --- | --- | --- | --- | --- | --- |
| **SNP** | **AGES** | **ARIC** | **CHS** | **FHS** | **RS** | **ARIC*** | **KORA F3** | **KORA F4** | **SHIP** |
| rs4072037 | 0.96 | 0.90 | 0.92 | 0.69 | 1.00 | 0.91 | 0.69 | 0.90 | 0.93 |
| rs13146355 | 0.98 | 0.95 | 0.95 | 0.96 | 0.97 | 0.96 | 0.94 | 0.94 | 0.96 |
| rs11144134 | 0.49 | 0.94 | 0.95 | 0.96 | 1.00 | 0.94 | 0.29 | 0.84 | 0.95 |
| rs3925584 | 0.94 | 1.00 | 0.83 | 1.00 | 1.00 | 1.00 | 0.99 | 0.98 | 0.99 |
| rs7965584 | 0.99 | 0.99 | 0.88 | 0.96 | 0.99 | 0.99 | 0.96 | 0.97 | 0.98 |
| rs7197653 | 0.84 | 0.87 | 0.79 | 0.80 | 0.88 | 0.87 | 0.83 | 0.86 | 0.88 |
| rs2592394 | 0.95 | 0.88 | 0.93 | 0.93 | 0.97 | 0.88 | 0.75 | 0.86 | 0.92 |
| rs448378 | 0.98 | 1.00 | 0.99 | 1.00 | 1.00 | 1.00 | 0.94 | 1.00 | 1.00 |
| rs4561213 | 0.97 | 0.97 | 0.88 | 0.93 | 0.95 | 0.92 | 0.83 | 0.93 | 0.95 |

AGES, Age, Gene/Environment Susceptibility--Reykjavik Study; ARIC, The Atherosclerosis Risk in Communities Study; CHARGE, Cohorts for Heart and Aging Research in Genomic Epidemiology; CHS, The Cardiovascular Health Study; FHS, The Framingham Heart Study; KORA, Kooperative Gesundheitsforschung in der Region Augsburg; RS. The Rotterdam Study; SHIP, The Study of Health in Pomerania; SNP, single nucleotide polymorphism.

*ARIC discovery and replication samples were independent of each other.
