# Supplementary material for: Genome-Wide Association Studies of Serum Magnesium, Potassium, and Sodium Concentrations Identify Six Loci Influencing Serum Magnesium Levels
Source: PLoS Genet. 2010 Aug 5;6(8):e1001045. doi: 10.1371/journal.pgen.1001045 (PMC2916845; doi:10.1371/journal.pgen.1001045)
Supplement: Table S8 — Association between systolic and diastolic blood pressure with the lead replicated SNPs showing genome-wide significant associations with serum magnesium concentrations in the CHARGE Consortium. (0.04 MB DOC) [file pgen.1001045.s010.doc]

**Table S8. Association between systolic and diastolic blood pressure with the lead replicated SNPs showing genome-wide significant associations with serum magnesium concentrations in the CHARGE Consortium.**

|  | **CHR** | **Closest Gene** | **N** | **Beta^*^** | **SE** | **P** |
| --- | --- | --- | --- | --- | --- | --- |
| Systolic Blood Pressure^†, ‡^ | | | | | | |
| rs4072037 | 1 | *MUC1* | 27,469 | 0.126 | 0.146 | 3.88E-01 |
| rs13146355 | 4 | *SHROOM3* | 27,469 | 0.233 | 0.144 | 1.04E-01 |
| rs11144134 | 9 | *TRPM6* | 27,469 | 0.134 | 0.272 | 6.22E-01 |
| rs3925584 | 11 | *DCDC5* | 27,469 | -0.142 | 0.143 | 3.20E-01 |
| rs7965584 | 12 | *ATP2B1* | 27,469 | 0.240 | 0.159 | 1.31E-01 |
| Diastolic Blood Pressure^‡, §^ | | | | | | |
| rs4072037 | 1 | *MUC1* | 27,647 | 0.104 | 0.089 | 2.41E-01 |
| rs13146355 | 4 | *SHROOM3* | 27,647 | 0.028 | 0.087 | 7.44E-01 |
| rs11144134 | 9 | *TRPM6* | 27,647 | -0.160 | 0.167 | 3.39E-01 |
| rs3925584 | 11 | *DCDC5* | 27,647 | -0.103 | 0.086 | 2.34E-01 |
| rs7965584 | 12 | *ATP2B1* | 27,647 | 0.114 | 0.096 | 2.34E-01 |

CHR, chromosome; SE, standard error

*adjusted for age, sex, study center, age squared, and body mass index; the association reported is for the magnesium-lowering allele

^†^Includes the following CHARGE cohorts: AGES (N=3,219), ARIC (N=8,047), CHS (N=3,370), FHS (N=8,096), RS (N=4,737); beta units are mmHg

^‡^For participants using hypertension medications, 10 mmHg was added to systolic blood pressure measures and 5 mmHg was added to diastolic blood pressure measures

^§^ Includes the following CHARGE cohorts: AGES (N=3,219), ARIC (N=8,047), CHS (N=3,368), FHS (N=8,096), RS (N=4,737); beta unites are mmHg
