# Supplementary material for: Genome-Wide Association Studies of Serum Magnesium, Potassium, and Sodium Concentrations Identify Six Loci Influencing Serum Magnesium Levels
Source: PLoS Genet. 2010 Aug 5;6(8):e1001045. doi: 10.1371/journal.pgen.1001045 (PMC2916845; doi:10.1371/journal.pgen.1001045)
Supplement: Text S1 — Supporting information. (0.10 MB DOC) [file pgen.1001045.s011.doc]

**Genome-wide Association Studies of Serum Magnesium, Potassium and Sodium Concentrations Identify Six Loci Influencing Serum Magnesium Levels**

Tamra E. Meyer, Germaine C. Verwoert, Shih-Jen Hwang, Nicole L. Glazer, Albert V. Smith, Frank J. A. van Rooij, Georg B. Ehret, Eric Boerwinkle, Janine F. Felix, Tennille S. Leak, Tamara B. Harris, Qiong Yang, Abbas Dehghan, Thor Aspelund, Ronit Katz, Georg Homuth, Thomas Kocher, Rainer Rettig, Janina S. Ried, Christian Gieger, Hanna Prucha, Arne Pfeufer, Thomas Meitinger, The Genetic Factors for Osteoporosis (GEFOS) Consortium, Meta Analysis of Glucose and Insulin Related Traits Consortium (MAGIC), Josef Coresh, Albert Hofman, Mark J. Sarnak, Yii-Der Ida Chen, André G. Uitterlinden, Aravinda Chakravarti, Bruce M. Psaty, Cornelia M. van Duijn, W. H. Linda Kao, Jacqueline C. M. Witteman, Vilmundur Gudnason, David S. Siscovick, Caroline S. Fox, Anna Köttgen

**Text S1**

**Table of Contents:**

1. Meta Analysis of Glucose and Insulin related traits Consortium (MAGIC)

contributing author list **Page 3**

1. The Genetic Factors for Osteoporosis (GEFOS) Consortium contributing

author list **Page 10**

**Meta Analysis of Glucose and Insulin related traits Consortium (MAGIC) contributing author list**

Josée Dupuis^1,2,178^, Claudia Langenberg^3,178^, Inga Prokopenko^4,5,178^, Richa Saxena^6,7,178^, Nicole Soranzo^8,9,178^, Anne U Jackson^10^, Eleanor Wheeler^11^, Nicole LGlazer^12^, Nabila Bouatia-Naji^13^, Anna LGloyn^4^, Cecilia MLindgren^4,5^, Reedik Mägi^4,5^, Andrew P Morris^5^, Joshua Randall^5^, Toby Johnson^14–16^, Paul Elliott^17,176^, Denis Rybin^18^, Gudmar Thorleifsson^19^, Valgerdur Steinthorsdottir^19^, Peter Henneman^20^, Harald Grallert^21^, Abbas Dehghan^22^, Jouke Jan Hottenga^23^, Christopher SFranklin^24^, Pau Navarro^25^, Kijoung Song^26^, Anuj Goel^5,27^, John R B Perry^28^, Josephine MEgan^29^, Taina Lajunen^30^, Niels Grarup^31^, Thomas Sparsø^31^, Alex Doney^32^, Benjamin F Voight^6,7^, Heather MStringham^10^, Man Li^33^, Stavroula Kanoni^34^, Peter Shrader^35^, Christine Cavalcanti-Proença^13^, Meena Kumari^36^, Lu Qi^37^, Nicholas J Timpson^38^, Christian Gieger^21^, Carina Zabena^39^, Ghislain Rocheleau^40,41^, Erik Ingelsson^42,43^, Ping An^44^, Jeffrey O’Connell^45^, Jian’an Luan^3^, Amanda Elliott^6,7^, Steven A McCarroll^6,7^, Felicity Payne^11^, Rosa Maria Roccasecca^11^, François Pattou^46^, Praveen Sethupathy^47^, Kristin Ardlie^48^, Yavuz Ariyurek^49^, Beverley Balkau^50^, Philip Barter^51^, John P Beilby^52,53^, Yoav Ben-Shlomo^54^, Rafn Benediktsson^55,56^, Amanda J Bennett^4^, Sven Bergmann^14,16^, Murielle Bochud^15^, Eric Boerwinkle^57^, Amélie Bonnefond^13^, Lori LBonnycastle^47^, Knut Borch-Johnsen^58,59^, Yvonne Böttcher^60^, Eric Brunner^36^, Suzannah J Bumpstead^8^, Guillaume Charpentier^61^, Yii-Der Ida Chen^62^, Peter Chines^47^, Robert Clarke^63^, Lachlan J MCoin^17^, Matthew NCooper^64^, Marilyn Cornelis^37^, Gabe Crawford^6^, Laura Crisponi^65^, Ian NMDay^38^, Eco J Cde Geus^23^, Jerome Delplanque^13^, Christian Dina^13^, Michael R Erdos^47^, Annette CFedson^64,66^, Antje Fischer-Rosinsky^67,68^, Nita GForouhi^3^, Caroline SFox^2,69^, Rune Frants^70^, Maria Grazia Franzosi^71^, Pilar Galan^72^, Mark OGoodarzi^62^, Jürgen Graessler^73^, Christopher J Groves^4^, Scott Grundy^74^, Rhian Gwilliam^8^, Ulf Gyllensten^75^, Samy Hadjadj^76^, Göran Hallmans^77^, Naomi Hammond^8^, Xijing Han^10^, Anna-Liisa Hartikainen^78^, Neelam Hassanali^4^, Caroline Hayward^25^, Simon CHeath^79^, Serge Hercberg^80^, Christian Herder^81^, Andrew A Hicks^82^, David R Hillman^66,83^, Aroon DHingorani^36^, Albert Hofman^22^, Jennie Hui^52,84^, Joe Hung^85,86^, Bo Isomaa^87,88^, Paul R V Johnson^4,89^, Torben Jørgensen^90,91^, Antti Jula^92^, Marika Kaakinen^93^, Jaakko Kaprio^94–96^, Y Antero Kesaniemi^97^, Mika Kivimaki^36^, Beatrice Knight^98^, Seppo Koskinen^99^, Peter Kovacs^100^, Kirsten Ohm Kyvik^101^, GMark Lathrop^79^, Debbie A Lawlor^38^, Olivier Le Bacquer^13^, Cécile Lecoeur^13^, Yun Li^10^, Valeriya Lyssenko^102^, Robert Mahley^103^, Massimo Mangino^9^, Alisa KManning^1^, María Teresa Martínez-Larrad^39^, Jarred B McAteer^6,104,105^, Laura J McCulloch^4^, Ruth McPherson^106^, Christa Meisinger^21^, David Melzer^28^, David Meyre^13^, Braxton DMitchell^45^, Mario A Morken^47^, Sutapa Mukherjee^66,83^, Silvia Naitza^65^, Narisu Narisu^47^, Matthew J Neville^4,107^, Ben A Oostra^108^, Marco Orrù^65^, Ruth Pakyz^45^, Colin NA Palmer^109^, Giuseppe Paolisso^110^, Cristian Pattaro^82^, Daniel Pearson^47^, John F Peden^5,27^, Nancy LPedersen^42^, Markus Perola^96,111,112^, Andreas F H Pfeiffer^67,68^, Irene Pichler^82^, Ozren Polasek^113^, Danielle Posthuma^23,114^, Simon CPotter^8^, Anneli Pouta^115^, Michael A Province^44^, Bruce MPsaty^116,117^, Wolfgang Rathmann^118^, Nigel WRayner^4,5^, Kenneth Rice^119^, Samuli Ripatti^96,111^, Fernando Rivadeneira^22,120^, Michael Roden^81,121^, Olov Rolandsson^122^, Annelli Sandbaek^123^, Manjinder Sandhu^3,124^, Serena Sanna^65^, Avan Aihie Sayer^125^, Paul Scheet^126^, Laura J Scott^10^, Udo Seedorf^127^, Stephen J Sharp^3^, Beverley Shields^98^, Gunnar Sigurðsson^55,56^, Eric J GSijbrands^22,120^, Angela Silveira^128^, Laila Simpson^64,66^, Andrew Singleton^129^, Nicholas LSmith^130,131^, Ulla Sovio^17^, Amy Swift^47^, Holly Syddall^125^, Ann-Christine Syvänen^132^, Toshiko Tanaka^133,134^, Barbara Thorand^21^, Jean Tichet^135^, Anke Tönjes^60,136^, Tiinamaija Tuomi^87,137^, André GUitterlinden^22,120^, Ko Willems van Dijk^70,138^, Mandy van Hoek^120^, Dhiraj Varma^8^, Sophie Visvikis-Siest^139^, Veronique Vitart^25^, Nicole Vogelzangs^140^, Gérard Waeber^141^, Peter J Wagner^96,111^, Andrew Walley^142^, GBragi Walters^19^, Kim LWard^64,66^, Hugh Watkins^5,27^, Michael NWeedon^28^, Sarah H Wild^24^, Gonneke Willemsen^23^, Jaqueline CMWitteman^22^, John WGYarnell^143^, Eleftheria Zeggini^5,8^, Diana Zelenika^79^, Björn Zethelius^43,144^, Guangju Zhai^9^, Jing Hua Zhao^3^, MCarola Zillikens^120^, DIAGRAMConsortium^145^, GIANTConsortium^145^, Global BPgen Consortium^145^, Ingrid B Borecki^44^, Ruth J F Loos^3^, Pierre Meneton^80^, Patrik KEMagnusson^42^, David MNathan^104,105^, Gordon H Williams^69,105^, Andrew THattersley^98^, Kaisa Silander^96,111^, Veikko Salomaa^146^, George Davey Smith^38^, Stefan R Bornstein^73^, Peter Schwarz^73^, Joachim Spranger^67,68^, Fredrik Karpe^4,107^, Alan R Shuldiner^45^, Cyrus Cooper^125^, George V Dedoussis^34^, Manuel Serrano-Ríos^39^, Andrew DMorris^109^, Lars Lind^132^, Lyle J Palmer^64,66,84^, Frank B Hu1^47,148^, Paul WFranks^149^, Shah Ebrahim^150^, Michael Marmot^36^, WH Linda Kao^33,151,152^, James SPankow^153^, Michael J Sampson^154^, Johanna Kuusisto^155^, Markku Laakso^155^, Torben Hansen^31,156^, Oluf Pedersen^31,59,157^, Peter Paul Pramstaller^82,158,159^, H Erich Wichmann^21,160,161^, Thomas Illig^21^, Igor Rudan^24,162,163^, Alan F Wright^25^, Michael Stumvoll^60^, Harry Campbell^24^, James F Wilson^24^, Anders Hamsten on behalf of Procardis Consortium^128^, Richard NBergman^164^, Thomas A Buchanan^164,165^, Francis SCollins^47^, Karen LMohlke^166^, Jaakko Tuomilehto^94,167, 168^, Timo TValle^167^, David Altshuler^6,7,104,105^, Jerome I Rotter^62^, David SSiscovick^169^, Brenda WJ H Penninx^140^, Dorret I Boomsma^23^, Panos Deloukas^8^, Timothy DSpector^8,9^, Timothy MFrayling^28^, Luigi Ferrucci^170^, Augustine Kong^19^, Unnur Thorsteinsdottir^19,171^, Kari Stefansson^19,171^, Cornelia Mvan Duijn^22^, Yurii SAulchenko^22^, Antonio Cao^65^, Angelo Scuteri^172,177^, David Schlessinger^47^, Manuela Uda^65^, Aimo Ruokonen^173^, Marjo-Riitta Jarvelin^17,93, 174^, Dawn MWaterworth^26^, Peter Vollenweider^141^, Leena Peltonen^8,48,96,111,112^, Vincent Mooser^26^, Goncalo R Abecasis^10^, Nicholas J Wareham^3^, Robert Sladek^40,41^, Philippe Froguel^13,142^, Richard MWatanabe^164,175^, James B Meigs^35,105^, Leif Groop^102^, Michael Boehnke^10^, Mark I McCarthy^4,5,107^, Jose CFlorez^6,7,104,105^ & Inês Barroso^11^ for the MAGIC investigators

^1^Department of Biostatistics, Boston University School of Public Health, Boston, Massachusetts, USA. ^2^National Heart, Lung, and Blood Institute’s Framingham Heart Study, Framingham, Massachusetts, USA. ^3^Medical Research Council (MRC), Epidemiology Unit, Institute of Metabolic Science, Addenbrooke’s Hospital, Cambridge, UK. ^4^Oxford Centre for Diabetes, Endocrinology and Metabolism, University of Oxford, Oxford, UK. ^5^Wellcome Trust Centre for Human Genetics, University of Oxford, Oxford, UK. ^6^Program in Medical and Population Genetics, Broad Institute, Cambridge, Massachusetts, USA. ^7^Center for Human Genetic Research, Massachusetts General Hospital, Boston, Massachusetts, USA. ^8^Wellcome Trust Sanger Institute, Hinxton, Cambridge, UK. ^9^Twin Research and Genetic Epidemiology Department, King’s College London, St Thomas’ Hospital Campus, London, UK. ^10^Center for Statistical Genetics, Department of Biostatistics, University of Michigan School of Public Health, Ann Arbor, Michigan, USA. ^11^Metabolic Disease Group, Wellcome Trust Sanger Institute, Hinxton, Cambridge, UK. ^12^Cardiovascular Health Research Unit and Department of Medicine, University of Washington, Seattle, Washington, USA. ^13^Centre National de la Recherche Scientifique–Unité Mixte de Recherche 8090, Pasteur Institute, Lille 2–Droit et Santé University, Lille, France. ^14^Department of Medical Genetics, University of Lausanne, Lausanne, Switzerland. ^15^University Institute of Social and Preventative Medicine, Centre Hospitalier Universitaire Vaudois (CHUV) and University of Lausanne, Lausanne, Switzerland. ^16^Swiss Institute of Bioinformatics, Lausanne, Switzerland. ^17^Department of Epidemiology and Public Health, Imperial College London, Faculty of Medicine, Norfolk Place, London, UK. ^18^Boston University Data Coordinating Center, Boston, Massachusetts, USA. ^19^deCODE Genetics, Reykjavik, Iceland. ^20^Department of Human Genetics, Leiden University Medical Centre, Leiden, The Netherlands. ^21^Institute of Epidemiology, Helmholtz Zentrum Muenchen, German Research Center for Environmental Health, Neuherberg, Germany. ^22^Department of Epidemiology, Erasmus Medical College, Rotterdam, The Netherlands. ^23^Department of Biological Psychology, VU University Amsterdam, Amsterdam, The Netherlands. ^24^Centre for Population Health Sciences, University of Edinburgh, Edinburgh, UK. ^25^MRC Human Genetics Unit, Institute of Genetics and Molecular Medicine, Edinburgh, UK. ^26^Division of Genetics, Research and Development, GlaxoSmithKline, King of Prussia, Pennsylvania, USA. ^27^Department of Cardiovascular Medicine, University of Oxford, Oxford, UK. ^28^Genetics of Complex Traits, Institute of Biomedical and Clinical Sciences, Peninsula College of Medicine and Dentistry, University of Exeter, Exeter, UK. ^29^National Institute of Aging, Baltimore, Maryland, USA. ^30^Unit for Child and Adolescent Health and Welfare, National Institute for Health and Welfare, Biocenter Oulu, University of Oulu, Oulu, Finland. ^31^Hagedorn Research Institute, Gentofte, Denmark. ^32^Department of Medicine and Therapeutics, Level 7, Ninewells Hospital and Medical School, Dundee, UK. ^33^Department of Epidemiology, Bloomberg School of Public Health, Johns Hopkins University, Baltimore, Maryland, USA. ^34^Department of Nutrition–Dietetics, Harokopio University, Athens, Greece. ^35^General Medicine Division, Massachusetts General Hospital, Boston, Massachusetts, USA. ^36^Department of Epidemiology and Public Health, University College London, London, UK. ^37^Departments of Nutrition and Epidemiology, Harvard School of Public Health, Boston, Massachusetts, USA. ^38^MRC Centre for Causal Analyses in Translational Epidemiology, University of Bristol, Bristol, UK. ^39^Fundación para la Investigación Biomédica del Hospital Clínico San Carlos, Madrid, Spain. ^40^Departments of Medicine and Human Genetics, McGill University, Montreal, Canada. ^41^Genome Quebec Innovation Centre, Montreal, Canada. ^42^Department of Medical Epidemiology and Biostatistics, Karolinska Institutet, Stockholm, Sweden. ^43^Department of Public Health and Caring Sciences, Uppsala University, Uppsala, Sweden. ^44^Division of Statistical Genomics, Department of Genetics, Washington University School of Medicine, St. Louis, Missouri, USA. ^45^Division of Endocrinology, Diabetes and Nutrition, University of Maryland School of Medicine, Baltimore, Maryland, USA. ^46^INSERM U859, Universite de Lille-Nord de France, Lille, France. ^47^Genome Technology Branch, National Human Genome Research Institute, Bethesda, Maryland, USA. ^48^The Broad Institute, Cambridge, Massachusetts, USA. ^49^Leiden Genome Technology Center, Leiden University Medical Center, Leiden, The Netherlands. ^50^INSERM U780, Paris Sud University, Villejuif, France. ^51^The Heart Research Institute, Sydney, New South Wales, Australia. ^52^PathWest Laboratory of Western Australia, Department of Molecular Genetics, J Block, QEII Medical Centre, Nedlands West Australia, Australia. ^53^School of Surgery and Pathology, University of Western Australia, Nedlands West Australia, Australia. ^54^Department of Social Medicine, University of Bristol, Bristol, UK. ^55^Landspitali University Hospital, Reykjavik, Iceland. ^56^Icelandic Heart Association, Kopavogur, Iceland. ^57^The Human Genetics Center and Institute of Molecular Medicine, University of Texas Health Science Center, Houston, Texas, USA. ^58^Steno Diabetes Center, Gentofte, Denmark. ^59^Faculty of Health Science, University of Aarhus, Aarhus, Denmark. ^60^Department of Medicine, University of Leipzig, Leipzig, Germany. ^61^Endocrinology–Diabetology Unit, Corbeil-Essonnes Hospital, Essonnes, France. ^62^Medical Genetics Institute, Cedars-Sinai Medical Center, Los Angeles, California, USA. ^63^Clinical Trial Service Unit and Epidemiological Studies Unit, University of Oxford, Oxford, UK. ^64^Centre for Genetic Epidemiology and Biostatistics, University of Western Australia, Perth, Australia. ^65^Istituto di Neurogenetica e Neurofarmacologia (INN), Consiglio Nazionale delle Ricerche, c/o Cittadella Universitaria di Monserrato, Monserrato, Cagliari, Italy. ^66^Western Australian Sleep Disorders Research Institute, Queen Elizabeth Medical Centre II, Perth, Australia. ^67^Department of Endocrinology, Diabetes and Nutrition, Charite-Universitaetsmedizin Berlin, Berlin, Germany. ^68^Department of Clinical Nutrition, German Institute of Human Nutrition Potsdam-Rehbruecke, Nuthetal, Germany. ^69^Division of Endocrinology, Diabetes, and Hypertension, Brigham and Women’s Hospital, Harvard Medical School, Boston, Massachusetts, USA. ^70^Department of Human Genetics, Leiden University Medical Centre, Leiden, The Netherlands. ^71^Department of Cardiovascular Research, Istituto di Ricerche Farmacologiche ‘Mario Negri’, Milan, Italy. ^72^Institut National de la Santé et de la Recherche Médicale, Institut National de la Recherche Agronomique, Université Paris 13, Bobigny Cedex, France. ^73^Department of Medicine III, Division Prevention and Care of Diabetes, University of Dresden, Dresden, Germany. ^74^Center for Human Nutrition, University of Texas Southwestern Medical Center, Dallas, Texas, USA. ^75^Department of Genetics and Pathology, Rudbeck Laboratory, Uppsala University, Uppsala, Sweden. ^76^Centre Hospitalier Universitaire, de Poitiers, Endocrinologie Diabetologie, CIC INSERM 0802, INSERM U927, Université de Poitiers, Unité de Formation et de Recherche, Médecine Pharmacie, Poitiers, France. ^77^Department of Public Health and Clinical Medicine, Section for Nutritional Research, Umeå University, Umeå, Sweden. ^78^Department of Clinical Sciences, Obstetrics and Gynecology, University of Oulu, University of Oulu, Finland. ^79^Centre National de Génotypage/Institut de génomique/Commissariat à l’énergie atomique, Evry Cedex, France. ^80^INSERM U872, Faculté de Médecine Paris Descartes, Paris Cedex, France. ^81^Institute for Clinical Diabetology, German Diabetes Center, Leibniz Center for Diabetes Research at Heinrich Heine University Düsseldorf, Düsseldorf, Germany. ^82^Institute of Genetic Medicine, European Academy Bozen/Bolzano (EURAC), Viale Druso, Bolzano, Italy, Affiliated Institute of the University Lübeck, Lübeck, Germany. ^83^Department of Pulmonary Physiology, Sir Charles Gairdner Hospital, Perth, Australia. ^84^Busselton Population Medical Research Foundation, Sir Charles Gairdner Hospital, Perth, Australia. ^85^Heart Institute of Western Australia, Sir Charles Gairdner Hospital, Nedlands West Australia, Australia. ^86^School of Medicine and Pharmacology, University of Western Australia, Nedlands West Australia, Australia. ^87^Folkhalsan Research Centre, Helsinki, Finland. ^88^Malmska Municipal Health Care Center and Hospital, Jakobstad, Finland. ^89^Nuffield Department of Surgery, University of Oxford, Oxford, UK. ^90^Research Centre for Prevention and Health, Glostrup University Hospital, Glostrup, Denmark. ^91^Faculty of Health Science, University of Copenhagen, Copenhagen, Denmark. ^92^National Institute for Health and Welfare, Unit of Population Studies, Turku, Finland. ^93^Institute of Health Sciences and Biocenter Oulu, University of Oulu, Oulu, Finland. ^94^Department of Public Health, Faculty of Medicine, University of Helsinki, Helsinki, Finland. ^95^National Institute for Health and Welfare, Unit for Child and Adolescent Mental Health, Helsinki, Finland. ^96^Institute for Molecular Medicine Finland (FIMM), University of Helsinki, Helsinki, Finland. ^97^Department of Internal Medicine and Biocenter Oulu, Oulu, Finland. ^98^Diabetes Genetics, Institute of Biomedical and Clinical Science, Peninsula College of Medicine and Dentistry, University of Exeter, Exeter, UK. ^99^National Institute for Health and Welfare, Unit of Living Conditions, Health and Wellbeing, Helsinki, Finland. ^100^Interdisciplinary Centre for Clinical Research, University of Leipzig, Leipzig, Germany. ^101^The Danish Twin Registry, Epidemiology, Institute of Public Health, University of Southern Denmark, Odense, Denmark. ^102^Department of Clinical Sciences, Diabetes and Endocrinology, Lund University, University Hospital Malmö, Malmö, Sweden. ^103^Gladstone Institute of Cardiovascular Disease, University of California, San Francisco, California, USA. ^104^Diabetes Research Center, Diabetes Unit, Massachusetts General Hospital, Boston, Massachusetts, USA. ^105^Department of Medicine, Harvard Medical School, Boston, Massachusetts, USA. ^106^Division of Cardiology, University of Ottawa Heart Institute, Ottawa, Ontario, Canada. ^107^Oxford National Institute for Health Research, Biomedical Research Centre, Churchill Hospital, Oxford, UK. ^108^Department of Clinical Genetics, Erasmus Medical College, Rotterdam, The Netherlands. ^109^Biomedical Research Institute, University of Dundee, Ninewells Hospital and Medical School, Dundee, UK. ^110^Department of Geriatric Medicine and Metabolic Disease, Second University of Naples, Naples, Italy. ^111^National Institute for Health and Welfare, Unit of Public Health Genomics, Helsinki, Finland. ^112^Department of Medical Genetics, University of Helsinki, Helsinki, Finland. ^113^Department of Medical Statistics, Epidemiology and Medical Informatics, Andrija Stampar School of Public Health, Medical School, University of Zagreb, Rockefellerova, Zagreb, Croatia. ^114^Department of Clinical Genetics, VU University and Medical Center, Amsterdam, The Netherlands. ^115^Department of Obstetrics and Gynaecology, Oulu University Hospital, Oulu, Finland. ^116^Departments of Medicine, Epidemiology and Health Services, University of Washington, Seattle, Washington, USA. ^117^Group Health Research Institute, Group Health Cooperative, Seattle, Washington, USA. ^118^Institute of Biometrics and Epidemiology, German Diabetes Centre, Leibniz Centre at Heinrich Heine University Düsseldorf, Düsseldorf, Germany. ^119^Department of Biostatistics, University of Washington, Seattle, Washington, USA. ^120^Department of Internal Medicine, Erasmus Medical College, Rotterdam, The Netherlands. ^121^Department of Metabolic Diseases, Heinrich Heine University Düsseldorf, Düsseldorf, Germany. ^122^Department of Public Health and Clinical Medicine, Section for Family Medicine, Umeå University, Umeå, Sweden. ^123^School of Public Health, Department of General Practice, University of Aarhus, Aarhus, Denmark. ^124^Department of Public Health and Primary Care, Strangeways Research Laboratory, University of Cambridge, Cambridge, UK. ^125^MRC Epidemiology Resource Centre, University of Southampton, Southampton General Hospital, Southampton, UK. ^126^Department of Epidemiology, University of Texas, M.D. Anderson Cancer Center, Houston, Texas, USA. ^127^Leibniz-Institut für Arterioskleroseforschung an der Universität Münster, Münster, Germany. ^128^Atherosclerosis Research Unit, Department of Medicine, Karolinska Institutet, Stockholm, Sweden. ^129^Laboratory of Neurogenetics, National Institute on Aging, Bethesda, Maryland, USA. ^130^Department of Epidemiology, University of Washington, Seattle, Washington, USA. ^131^Seattle Epidemiologic Research and Information Center, Department of Veterans Affairs Office of Research and Development, Seattle, Washington, USA. ^132^Department of Medical Sciences, Uppsala University, Uppsala, Sweden. ^133^Medstar Research Institute, Baltimore, Maryland, USA. ^134^Clinical Research Branch, National Institute on Aging, Baltimore, Maryland, USA. ^135^Institut interrégional pour la santé (IRSA), La Riche, France. ^136^Coordination Centre for Clinical Trials, University of Leipzig, Leipzig, Germany. ^137^Department of Medicine, Helsinki University Hospital, University of Helsinki, Helsinki, Finland. ^138^Department of Internal Medicine, Leiden University Medical Centre, Leiden, The Netherlands. ^139^Research Unit, Cardiovascular Genetics, Nancy University Henri Poincaré, Nancy, France. ^140^EMGO Institute for Health and Care Research, Department of Psychiatry, VU University Medical Center, Amsterdam, The Netherlands. ^141^Department of Internal Medicine, Centre Hospitalier Universitaire Vaudois, Lausanne, Switzerland. ^142^Genomic Medicine, Imperial College London, Hammersmith Hospital, London, UK. ^143^Epidemiology and Public Health, Queen’s University Belfast, Belfast, UK. ^144^Medical Products Agency, Uppsala, Sweden. ^145^See Supplementary Note for a full list of authors. ^146^National Institute for Health and Welfare, Unit of Chronic Disease Epidemiology and Prevention, Helsinki, Finland. ^147^Departments of Nutrition and Epidemiology, Harvard School of Public Health, Boston, Massachusetts, USA. ^148^Channing Laboratory, Brigham and Women’s Hospital and Harvard Medical School, Boston, Massachusetts, USA. ^149^Genetic Epidemiology and Clinical Research Group, Department of Public Health and Clinical Medicine, Section for Medicine, Umeå University Hospital, Umeå, Sweden. ^150^London School of Hygiene and Tropical Medicine, London, UK. ^151^Department of Medicine, School of Medicine, Johns Hopkins University, Baltimore, Maryland, USA. ^152^The Welch Center for Prevention, Epidemiology, and Clinical Research, School of Medicine and Bloomberg School of Public Health, Johns Hopkins University, Baltimore, Maryland, USA. ^153^Division of Epidemiology and Community Health, School of Public Health, University of Minnesota, Minneapolis, Minnesota, USA. ^154^Department of Endocrinology and Diabetes, Norfolk and Norwich University Hospital National Health Service Trust, Norwich, UK. ^155^Department of Medicine, University of Kuopio and Kuopio University Hospital, Kuopio, Finland. ^156^Faculty of Health Science, University of Southern Denmark, Odense, Denmark. ^157^Institute of Biomedical Science, Faculty of Health Science, University of Copenhagen, Copenhagen, Denmark. ^158^Department of Neurology, General Central Hospital, Bolzano, Italy. ^159^Department of Neurology, University of Lübeck, Lübeck, Germany. ^160^Institute of Medical Informatics, Biometry and Epidemiology, Ludwig-Maximilians-Universität, Munich, Germany. ^161^Klinikum Grosshadern, Munich, Germany. ^162^School of Medicine, University of Split, Split, Croatia. ^163^Gen-Info Ltd., Zagreb, Croatia. ^164^Department of Physiology and Biophysics, Keck School of Medicine, University of Southern California, Los Angeles, California, USA. ^165^Department of Medicine, Division of Endocrinology, Keck School of Medicine, University of Southern California, Los Angeles, California, USA. ^166^Department of Genetics, University of North Carolina, Chapel Hill, North Carolina, USA. ^167^National Institute for Health and Welfare, Unit of Diabetes Prevention, Helsinki, Finland. ^168^South Ostrobothnia Central Hospital, Seinajoki, Finland. ^169^Departments of Medicine and Epidemiology, University of Washington, Seattle, Washington, USA. ^170^Longitudinal Studies Section, Clinical Research Branch, National Institute on Aging, NIH, Baltimore, Maryland, USA. ^171^Faculty of Medicine, University of Iceland, Reykjavík, Iceland. ^172^Lab of Cardiovascular Sciences, National Institute on Aging, National Institutes of Health, Baltimore, Maryland, USA. ^173^Department of Clinical Sciences/Clinical Chemistry, University of Oulu, University of Oulu, Oulu, Finland. ^174^National Institute of Health and Welfare, Oulu, Finland. ^175^Department of Preventive Medicine, Keck School of Medicine, University of Southern California, Los Angeles, California, USA. ^176^MRC–Health Protection Agency Centre for Environment and Health, Imperial College London, London, UK. ^177^UOC Geriatria, Istituto Nazionale Ricovero e cura per Anziani (INRCA) IRCCS, Rome, Italy. ^178^These authors contributed equally to this work. Correspondence should be addressed to M.B. (boehnke@umich.edu), M.I.M. (mark.mccarthy@drl.ox.ac.uk), J.C.F. (jcflorez@partners.org) or I.B. (ib1@sanger.ac.uk).

**The Genetic Factors for Osteoporosis (GEFOS) Consortium contributing author list**

Fernando Rivadeneira,^1,2,19^ Unnur Styrkársdottir,^3,19^ Karol Estrada,^1,19^ Bjarni V. Halldórsson,^3,4,19^ Yi-Hsiang Hsu,^5,19^ J. Brent Richards,^6,7,8,19^ M. Carola Zillikens,^1,19^ Fotini K. Kavvoura,^9,19^ Najaf Amin,^2^ Yurii S. Aulchenko,^2^ L. Adrienne Cupples,^10^ Panagiotis Deloukas,^11^ Serkalem Demissie,^10^ Elin Grundberg,^7,12^ Albert Hofman,^2^ Augustine Kong,^3^ David Karasik,^5^ Joyce B. van Meurs,^1^ Ben Oostra,^13^ Tomi Pastinen,^7,12^ Huibert A.P. Pols,^1,2^ Gunnar Sigurdsson,^14,15^ Nicole Soranzo,^11,6^ Gudmar Thorleifsson,^3^ Unnur Thorsteinsdottir,^3,14^ Frances MK Williams,^8^ Scott G. Wilson,^8,16^ Yanhua Zhou,^10^ Stuart H. Ralston,^17^ Cornelia M. van Duijn,^2,19^ Timothy Spector,^8,19^ Douglas P. Kiel,^5,19^ Kari Stefansson,^3,14,19^ John P.A. Ioannidis,^9,18,19^ André G. Uitterlinden,^1,2,19^ and the GEnetic Factors For Osteoporosis (GEFOS) Consortium

^1^Department of Internal Medicine, Erasmus MC, Rotterdam, 3015GE, The Netherlands, ^2^Department of Epidemiology, Erasmus MC, Rotterdam, 3015GE, The Netherlands, ^3^deCODE Genetics, 101 Reykjavík, Iceland, ^4^Reykjavik University, 103 Reykjavik, Iceland, ^5^Hebrew SeniorLife, Harvard Medical School, Boston, MA, 02131 USA, ^6^Department of Medicine, McGill University, Montréal, H3G 1Y6 Canada, ^7^Department of Human Genetics, McGill University, Montréal, H3G 1Y6 Canada, ^8^Department of Twin Research and Genetic Epidemiology, Kings College London, London, SE1 7EH, United Kingdom, ^9^Department of Hygiene and Epidemiology, University of Ioannina School of Medicine, Ioannina 45110, Greece, ^10^Department of Biostatistics, School of Public Health, Boston University, Boston, MA, 02118 USA, ^11^Wellcome Trust Sanger Institute, Hinxton, Cambridge, CB10 1SA, UK, ^12^McGill University and Genome Quebec Innovation Centre, Montreal, H3A 1A4, Canada, ^13^Department of Clinical Genetics, Erasmus MC, Rotterdam, 3015GE, The Netherlands, ^14^Faculty of Medicine, University of Iceland, 101 Reykjavík, Iceland, ^15^Department of Endocrinology and Metabolism, University Hospital, 108 Reykjavik, Iceland, ^16^School of Medicine & Pharmacology, The University of Western Australia and Department of Endocrinology & Diabetes, Sir Charles Gairdner Hospital, Nedlands, Western Australia, ^17^Rheumatic Diseases Unit, Institute of Genetics and Molecular Medicine, Western General Hospital, University of Edinburgh, Edinburgh, EH4 2XU, United Kingdom, ^18^Center for Genetic Epidemiology and Modeling, Tufts Medical Center, Tufts University School of Medicine, Boston, MA 02111, USA, ^19^These authors contributed equally to this work. Correspondence should be addressed to: Prof. Dr. André G. Uitterlinden, Genetic Laboratory - Room Ee 575, Department of Internal Medicine, Erasmus MC, PO Box 2040, NL-3000-DR Rotterdam, The Netherlands, (; Email: [a.g.uitterlinden@erasmusmc.nl](mailto:a.g.uitterlinden@erasmusmc.nl)
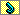
) & Prof. Dr. John P. Ioannidis, Department of Hygiene and Epidemiology, University of Ioannina School of Medicine, PO Box 1186, 45110 Ioannina, Greece, (; Email: [jioannid@cc.uoi.gr](mailto:jioannid@cc.uoi.gr)
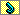
).
